# Real-Time Imaging of HIV-1 Protease Activation and Substrate Cleavage in Single Virions Assembling on the Plasma Membrane

**DOI:** 10.3390/v18080819

**Published:** 2026-07-25

**Authors:** Yinglin Li, Satya P. Singh, Mariana Marin, Alec L. Zhan, Ashwanth C. Francis, Gregory B. Melikyan

**Affiliations:** 1 Department of Pediatrics, Division of Infectious Diseases, Emory University School of Medicine, Atlanta, GA 30322, USA; 2 Institute of Molecular Biophysics and Department of Biological Sciences, Florida State University, Tallahassee, FL 32306, USA; ss22co@fsu.edu (S.P.S.); acfrancis@bio.fsu.edu (A.C.F.); 3 Children’s Healthcare of Atlanta, Atlanta, GA 30322, USA

**Keywords:** HIV-1 assembly, single-virus tracking, HIV-1 protease activation, substrate cleavage by viral protease, Förster resonance energy transfer

## Abstract

The timing of HIV-1 protease (PR) activation and the determinants of orderly Gag and Gag-Pol polyprotein cleavage, leading to structural maturation of virions, are not well-understood. Here, we employed total internal reflection microscopy to visualize PR activation and cleavage of a FRET-based fluorescent substrate in single HIV-1 particles assembled on the cell’s ventral membrane. PR activity manifested as a reduction in FRET signal in a relatively small fraction of nascent viral particles. By contrast, most virions released from cells contained a processed FRET substrate, suggesting that PR activation may be delayed or suppressed in most virions assembled at the ventral membrane. A combination of single-particle tracking and FRET measurements detected PR activity, on average, within ~20 min after the onset of particle assembly, while cleavage of a FRET substrate in single virions was completed within a few minutes. Importantly, substrates containing distinct PR cleavage sequences derived from Gag and Gag-Pol polyproteins were processed with different rates and efficiency. Our findings validate the use of surrogate FRET substrates to assess the timing of PR activation in single virions and elucidate the determinants of the tightly orchestrated order of Gag and Gag-Pol cleavage required for the formation of infectious virions.

## 1. Introduction

HIV-1 is released from the plasma membrane of infected cells as a non-infectious immature particle containing unprocessed Gag and Gag-Pol polyproteins (reviewed in [1,2]). Gag consists of the matrix (MA), capsid (CA), nucleocapsid (NC) and p6 domains interspersed with the SP1 and SP2 spacer peptides. The p6 domain recruits the cellular ESCRT (endosomal sorting complexes required for transport) machinery that drives virus budding from the plasma membrane. The Gag-Pol precursor consists of Gag, including the viral enzymes, protease (PR), reverse transcriptase (RT), integrase (IN), and the trans-frame peptide (TF). Gag-Pol is expressed and incorporated into virions in a ~1:20 ratio to Gag polyprotein [3,4]. At some point during or after virus budding from a cell, the PR autoactivates and proceeds to cleave distinct sites in Gag and Gag-Pol in a defined order that is critical to the formation of the mature cone-shaped HIV-1 capsid [5,6].

The cleavage order of Gag in virions is reported to be: SP1/NC → SP2/p6 ≥ MA/CA → NC/SP2 ≥ CA/SP1 [4,6,7,8]. Insights into the rate of PR-mediated Gag/Gag-Pol cleavage have been gained using surrogate Gag or Gag-Pol derived substrates in vitro. These experiments have shown that processing rates are modulated by the cleavage sequence and structural context of Gag and Gag-Pol polyprotein assemblies [8,9,10,11,12]. Indeed, the processing rate of certain cleavage sites in vitro does not appear to match the order of Gag processing in virions [4,6,7,8,11,12]. These results warrant investigation of relative cleavage rates of distinct Gag/Gag-Pol cleavage sequences in virions, in the same structural context.

The timing of PR autoactivation is critical for HIV-1 maturation. Premature activation of PR reduces the release of mature virions and activates the inflammasome sensor, CARD8, thereby inducing cell death *via* pyroptosis [13,14,15,16]. Delayed PR activation has also been shown to be deleterious for HIV-1 infectivity, leading to the production of morphologically defective particles with anomalous capsid architectures and aberrant RT processing patterns [17,18]. Yet, the timing of HIV-1 PR autoactivation in immature particles remains poorly characterized (reviewed in [4,5,6,19]). HIV-1 PR is a constitutive dimer that has broad specificity allowing it to cleave divergent sequences linking the individual Gag and Gag-Pol domains. It is believed that PR autoprocessing is initiated through Gag-Pol dimerization and involves two sequential cleavage events—intramolecular cleavage at the TF/PR site (also denoted p6Pol/PR) and intermolecular cleavage at the PR/RT site [20,21,22]. Supporting the notion that PR is autoactivated upon Gag-Pol dimerization are the findings that (i) the increased Gag-Pol/Gag ratio causes premature PR activation [23,24] and (ii) certain non-nucleoside RT inhibitors (NNRTIs) trigger premature PR activation by promoting Gag-Pol dimerization [13,14,15,16,25]. This model is also consistent with the slow rearrangement of immature Gag lattice in virions, which may be rate-limiting for Gag-Pol dimerization [26].

The timing of PR autoactivation remains controversial, with conflicting results reported using different PR activity assays. The timing of PR activation ranges from before [27] to a few seconds [28], several minutes [29], or even ~1 h [30] after HIV-1 budding. Bulk cell-based assays used to study PR autoactivation suffer from the highly asynchronous HIV-1 release from infected cells. Attempts to synchronize PR activation after HIV-1 release failed to produce infectious HIV-1 [17,18] and, thus, do not fully capture on-path Gag/Gag-Pol processing in budding virions. To circumvent this limitation, PR activity in single assembling HIV-1 particles has been imaged using Gag-based FRET (Förster resonance energy transfer) probes [29,31]. However, while these measurements afforded the time-resolved tracking of PR activity in single virions, Gag-fluorescent protein fusions have been shown to disrupt viral assembly [27,30,32]. In this regard, the use of Vpr-based FRET probes to monitor HIV-1 assembly and PR activation appears less disruptive [27,33,34]. However, these measurements were performed using flow virometry [27,35], which lacks adequate temporal resolution to detect substrate cleavage at single-virus resolution in real time.

Here, we tracked the assembly of single viruses containing a Vpr-based FRET substrate that enabled the real-time detection of PR activity through a reduction in FRET signal. Using this approach, we observed PR activation and surrogate substrate cleavage in a small fraction of virions assembled on the ventral membrane of cells. In control experiments, no changes in FRET signal were detected in the presence of the HIV-1 PR inhibitor, saquinavir (SQV). The timing of PR activation and the lack of release of fluorescence acceptor into the cytoplasm, following its liberation from a FRET substrate by PR, suggest that PR is activated shortly after virus budding from cells. Tracking the reduction in FRET signal from single virions showed that substrate cleavage within single virions was typically completed within a few minutes. We inserted distinct PR cleavage sites from the Gag and Gag-Pol domains into a Vpr-based FRET probe and assessed their processing in single virions. The distinct cleavage sequences modulated the rate and extent of PR-mediated substrate processing. We found that unlike other sequences tested, the sequences flanking the PR domain of Gag-Pol were cleaved with the apparent first-order kinetics and that the p6Pol/PR sequence N-terminal to PR was the most resistant to cleavage. These results support the validity of our single-virus imaging approach to assess the timing of HIV-1 PR activation and the rates of processing of distinct cleavage sequences linking individual Gag and Gag-Pol domains in the context of a surrogate substrate. 

## 2. Results

### 2.1. Visualization of Substrate Cleavage by HIV-1 Protease in Newly Assembled Single Virions

**Figure 1:**
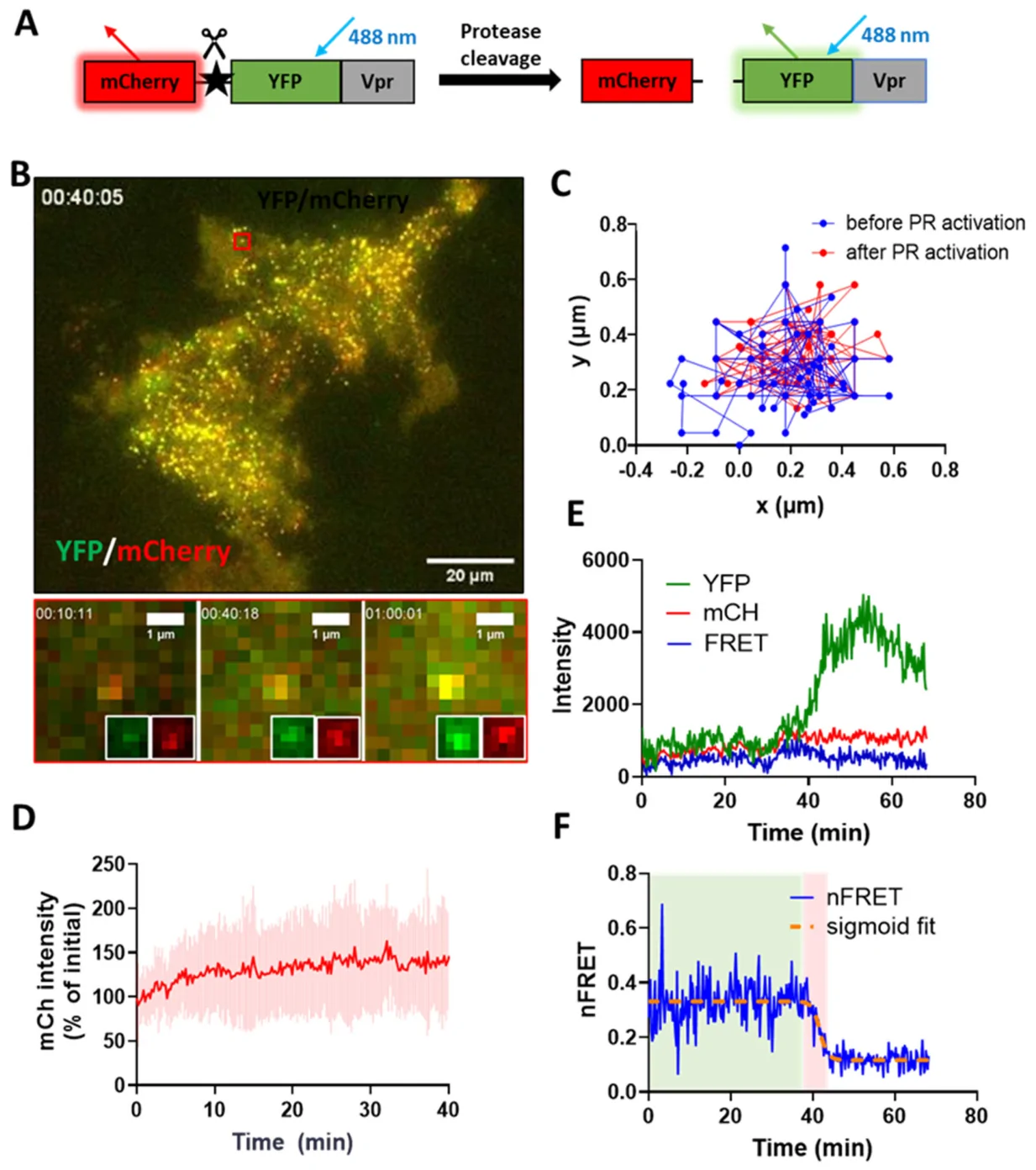
Live-cell imaging of HIV-1 protease activation in single nascent virions. (**A**) A drawing of mCherry-2xcl-YFP-Vpr FRET substrate for HIV-1 protease (PR) in which mCherry and YFP are linked *via* an HIV-1 protease cleavable linker (marked by scissors). Cleavage of this construct results in disruption of FRET. (**B**) HeLa cells transiently expressing HIV-1 backbone, HXB2 Env, and mCherry-2xcl-YFP-Vpr (43 h post-transfection (hpt)) were imaged in TIRF mode. A representative cell with multiple HIV-1 assembly sites is shown. YFP is colored green, and mCherry is red. *Inset*: An example of a nascent particle exhibiting reduction in FRET signal (corresponding to the boxed area in (**B**)). Snapshots taken 10, 40 and 60 min after initial appearance of a viral assembly site are shown (see also Movies S1 and S2). Adjacent particles not exhibiting decrease in nFRET are shown in Movie S3. (**C**) Motion trajectory of the particle in B before and after PR activation. (**D**) Increase in average mCherry intensity of nascent viral particles after initial signal appearance. Fifty individual mCherry intensity traces after background subtraction were aligned at the time of appearance, and the average intensity at every time point was calculated. Error bars (pink vertical lines) are SDs. (**E**) Fluorescence intensity traces obtained by tracking the particle shown in (**A**). The increase in YFP (donor, green) signal and concomitant reduction in FRET channel (blue line) at ~40 min indicate cleavage of the linker between YFP and mCherry. The FRET channel represents sensitized emission of mCherry resulting from a non-radiative energy transfer from YFP excited by 488 nm light. (**F**) A normalized FRET (nFRET) trace for the particle shown in (**A**) fitted with a sigmoidal function (dashed line; see Section 4.6). Green background segment indicates the time from onset of particle assembly to initial drop in nFRET; pink background segment marks the transition time from high to low nFRET.

To indirectly monitor HIV-1 PR activity in assembled virions, we and others have used Vpr-based FRET sensors, consisting of the N-terminal Vpr fusion with a FRET donor/acceptor pair linked by a PR cleavage sequence [27,36]. We have previously engineered the mCherry-2xcl-YFP-Vpr construct by inserting a chimeric PR cleavage sequence (SQNY/PIVQN-GGR-IRKVL, termed 2xcl) between mCherry and YFP-Vpr (Figure 1A) [36]. The chimeric PR cleavage sequence was originally used to improve the substrate processing efficiency. We have shown that PR-mediated cleavage of the 2xcl site in mCherry-2xcl-YFP-Vpr is manifested by a reduced FRET signal in cell-free virions due to liberation of mCherry. Free mCherry remains trapped within the viral membrane and is released upon its permeabilization with saponin or as a result of virus–cell fusion [36]. We chose this Vpr-based probe over direct Gag labeling to monitor PR activity in single budding virions for the following reasons. First, labeling Gag with a FRET pair is likely to disrupt viral assembly and/or maturation [27,30,32]. Second, cleavage of a Vpr-based FRET substrate has been shown to strongly correlate with the extent of Gag processing in HIV-1 particles [27,35]. Finally, and surprisingly, fluorescent Vpr spots forming at the plasma membrane appear to better report HIV-1 assembly sites than puncta formed by fluorescently tagged Gag [33].

We thus wanted to investigate if mCherry-2xcl-YFP-Vpr accumulates at the sites of viral assembly on the plasma membrane through its recruitment by Gag and if the subsequent PR activation at these sites can be detected by a decrease in FRET between YFP and mCherry. HeLa cells grown in a glass-bottom 35 mm cell culture dish were transfected with the plasmids expressing the HIV-1 structural proteins and mCherry-2xcl-YFP-Vpr (see Section 4). Total internal reflection (TIRF) microscopy revealed asynchronous formation and accumulation of mCherry/YFP-positive puncta at the ventral membrane of cells imaged between 24 and 48 h after transfection (Figure 1B). TIRF imaging minimizes a strong signal from mCherry-2xcl-YFP-Vpr in the cytoplasm, thereby enabling the visualization of recruitment of this marker by Gag/Gag-Pol at the sites of single HIV-1 assembly on the ventral membrane.

Single-virus tracking (Figure 1B,C) was employed to follow the appearance of discrete fluorescent puncta at the ventral membrane and subsequent changes in FRET signal. The intensity of YFP and mCherry signals from single puncta increased over time, likely reflecting the recruitment of fluorescent Vpr to Gag/Gag-Pol assembly sites (Figure 1D). Nearly all assembling particles exhibited limited mobility (Figure 1C). In these experiments, the imaging window could not be consistently extended beyond 2 h due to the crowding of assembling pseudoviruses on the plasma membrane which precluded subsequent single-particle tracking (Figure 1B). This was, in large part, because the assembled particles were rarely released from the ventral cell membrane. The lack of virus release from the ventral membrane should not be due to expression of BST-2/tetherin in HeLa cells [37], since cells were transfected with a Vpu-encoding HIV-1 vector which should downregulate tetherin [38,39]. In addition, particle crowding was observed on producer HEK293T cells lacking BST-2/tetherin (Supplementary Figure S1A). 

Importantly, for a fraction of fluorescent YFP/mCherry spots at the ventral membrane, we observed a marked increase in YFP signal and reduction in sensitized mCherry emission (emission upon excitation with a 488 nm laser) due to FRET, without a notable change in fluorescence of mCherry directly excited with a 561 nm laser (Figure 1E and Movies S1 and S2). These changes are hallmarks of diminished FRET between YFP and mCherry [36]. Indeed, the calculated normalized FRET (nFRET) values (see Section 4) for this subset of nascent particles revealed a significant reduction in nFRET (Figure 1F) coinciding with the rise of YFP signal (Figure 1E,F). Given the relatively quick drop in nFRET signal following a pronounced lag after the onset of particle assembly, this decrease was interpreted as a PR activation event, likely occurring after virus budding. Interestingly, assembled virions did not usually exhibit an increase in mobility after the drop in FRET signal (Figure 1C), suggesting that virions remain tethered to the plasma membrane after budding.

### 2.2. Decrease in Single-Particle FRET Signal Is Linked to PR Activity

**Figure 2:**
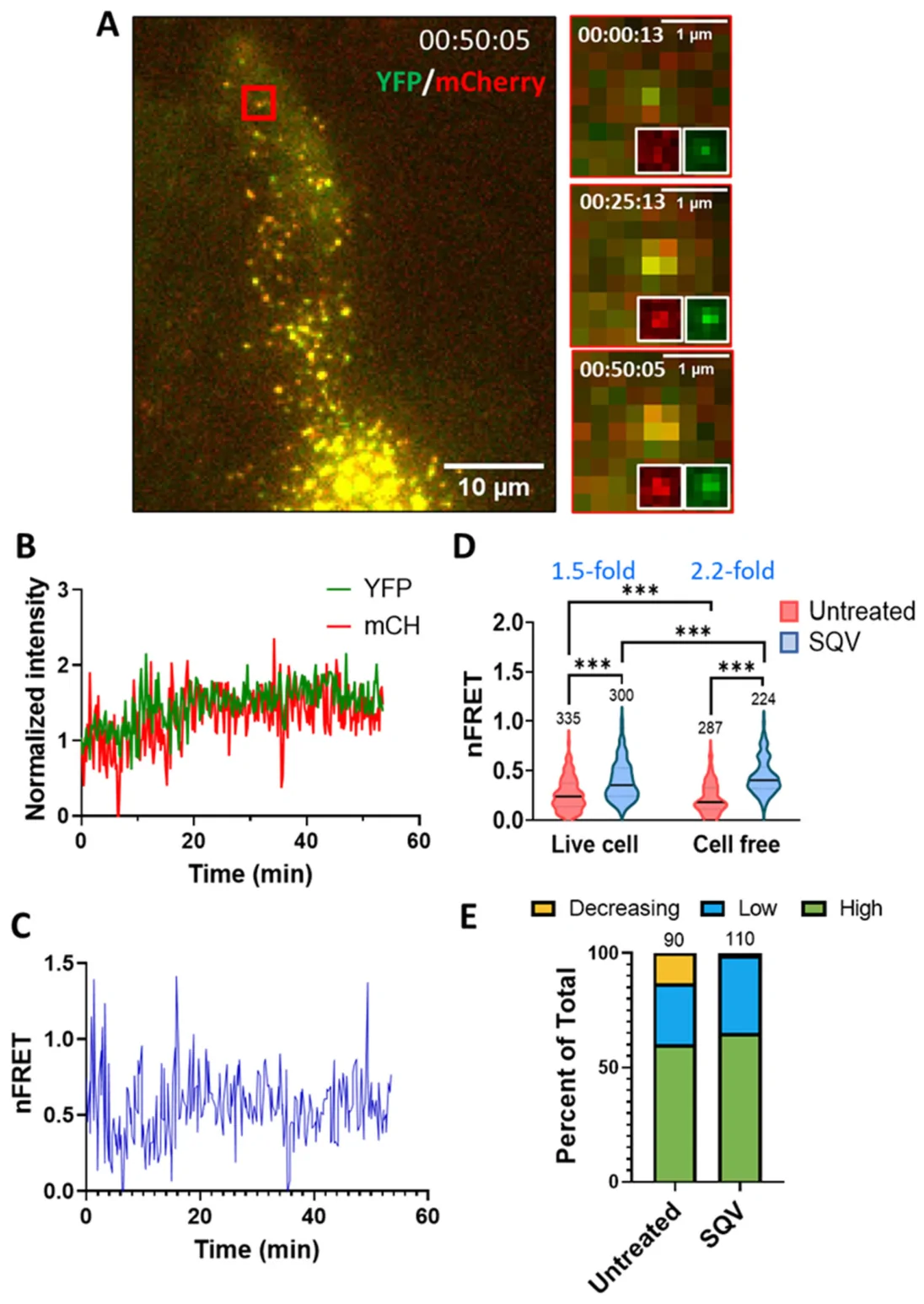
HIV-1 protease activation is not detected in the presence of a PR inhibitor. (**A**) TIRF imaging of HeLa cells 43 h post-transfection with mCherry-2xcl-YFP-Vpr and requisite plasmids, as in Figure 1, but in the presence of 500 nM saquinavir (SQV). *Inset*: A close-up view of the randomly selected viral particle (red box). Snapshots taken 0, 25, and 50 min after the initial appearance of the particle are shown. (**B**) Fluorescence intensity traces corresponding to the boxed viral particle in A. The sensitized mCherry emission channel is not included for clarity. (**C**) A normalized FRET trace for the virus boxed in A. (**D**) The distribution of nFRET values for cell-free viruses and nascent cell-associated particles produced in the absence or in the presence of SQV shows a statistically significant difference between mature and immature virus populations (***, *p* < 0.001). SQV treatment increased the median nFRET of cell-associated and cell-free virions 1.5- and 2.2-fold, respectively. Cell-free viruses were produced in 293T cells. The number of particles analyzed is shown above the plots. (**E**) Randomly selected newly forming particles in the presence or absence of SQV were tracked and categorized based upon their nFRET values as low or high or as particles exhibiting a decrease in nFRET (decreasing). nFRET > 0.25 was considered high, while nFRET < 0.25 was considered low. The nFRET cut-off value was set based upon the range of nFRET observed in the SQV-treated samples. The number of particles analyzed is shown above the plots.

To verify that the observed reduction in single-virus nFRET signal is a result of PR activity, we conducted control experiments in the presence of the PR inhibitor, saquinavir (SQV; Figure 2). As expected, SQV virtually abrogated the occurrence of decreasing nFRET in assembled virions (Figure 2A–C), except for one particle out of 110 analyzed (Supplementary Figure S2 and Movie S3). Additionally, the average nFRET values for virions assembled on the plasma membrane or released by cells into the medium were significantly higher in the presence of SQV relative to untreated samples (Figure 2D). The lower average nFRET value and the more pronounced effect of SQV treatment for cell-free (2.2-fold) *vs.* cell-associated (1.5-fold) virions (Figure 2D) suggest that a larger fraction of released virions (in the absence of SQV) contain a cleaved FRET substrate compared with nascent particles at the ventral membrane. These results imply that the nFRET drop after HIV-1 assembly reports the PR activity in nascent virions and that within our imaging window, PR activation occurs in a relatively small fraction of the newly formed particles on the ventral membrane of HeLa cells.

We next wanted to determine if visual inspection of particles at the ventral membrane may miss the PR activation events due to a poor signal/background ratio. Indeed, tracking randomly selected nascent particles, in addition to those exhibiting a clear increase in YFP signal, revealed occasional virions exhibiting a subtle decrease in nFRET signal (Supplementary Figure S3). Together with visually identified PR activation events, 13.3% of all preexisting or newly formed particles at the ventral membrane exhibited a decrease in nFRET within the 2 h imaging window (Figure 2E). We further analyzed the distribution of initial nFRET values of assembling particles. For this, we categorized nascent particles as exhibiting low (<0.25) and high (>0.25) nFRET, based upon the nFRET value distribution for particles assembling in the presence of SQV (Supplementary Figure S4). This analysis revealed that surprisingly, 26.7% of particles exhibited a low initial nFRET signal below 0.25 that did not change over the course of imaging (Figure 2E and Figure S5). The rest of nascent particles exhibited a stable, high nFRET signal (above 0.25, on average). A similar distribution of nFRET values was seen for HIV-1 particles forming on the ventral membrane of HEK293T cells (Figure S1E). The nature of a low initial nFRET signal from nascent virions is not understood (see Section 3). In summary, the relatively low fraction of particles exhibiting a drop in nFRET signal indicates that PR activation in most particles released from the ventral membrane may be delayed relative to our observation window.

### 2.3. Vpr-Based FRET Substrate Enables Quantitative Assessment of PR Activity in Single Virions

**Figure 3:**
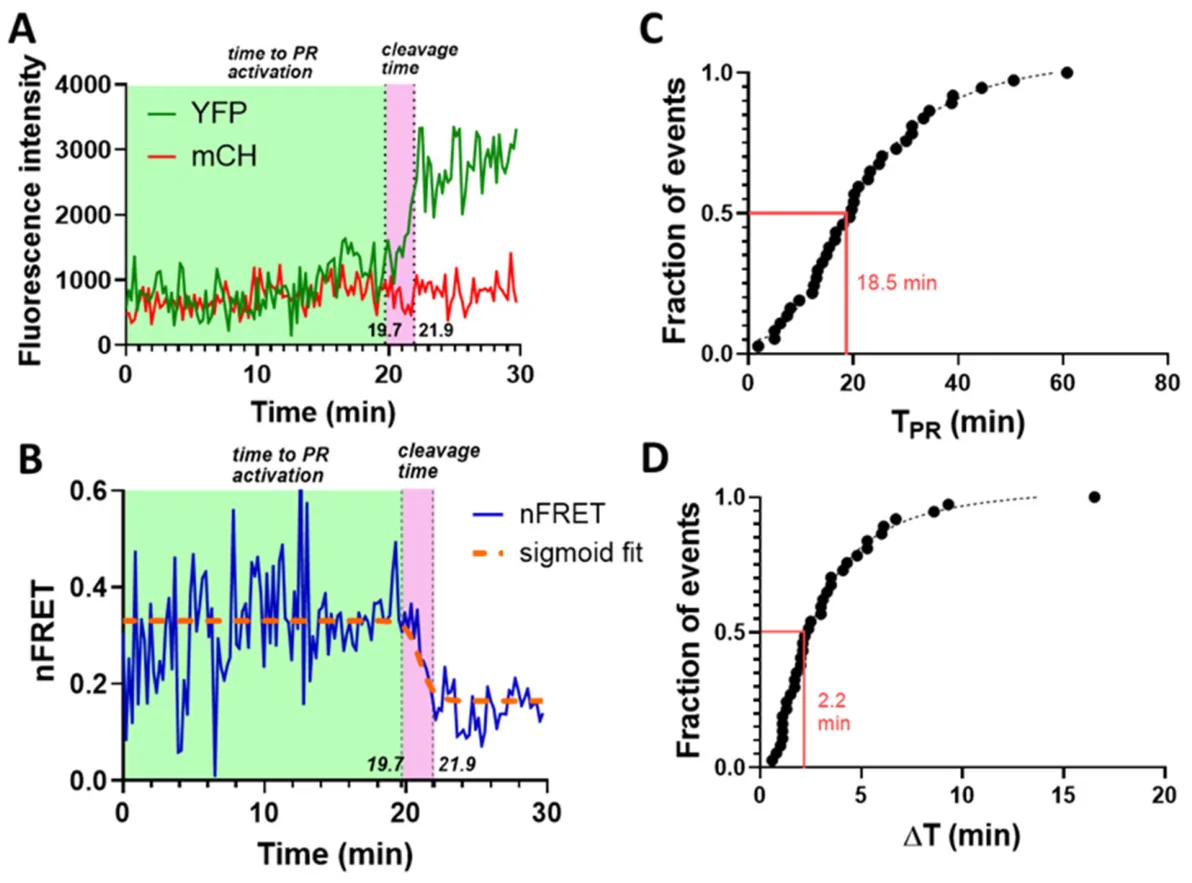
Measurements of PR activation and substrate cleavage rates in single HIV-1 particles. (**A**) Representative intensity traces show an increase in YFP signal (excited by a 488 nm laser) relative to mCherry signal (excited by a 561 nm laser), indicating protease-mediated cleavage of the mCherry-2xcl-YFP-Vpr FRET substrate. (**B**) nFRET trace calculated for the particle in panel (**A**) (see Section 4) was fitted with a sigmoidal function (dashed line). The time interval shaded in green corresponds to the waiting time from the onset of particle assembly to protease activation (detectable drop in nFRET). The drop in nFRET signal highlighted in pink corresponds to the time to substrate cleavage (nFRET plateauing at the lower level). The onset and completion of PR activity are defined as 10% and 90% drops in nFRET signal, respectively. (**C**,**D**) Cumulative distributions of waiting times for PR activation (**C**) and substrate cleavage duration (**D**) for the FRET substrate with the 2xcl cleavage site, yielding half-times of 18.5 min and 2.2 min, respectively. Dashed lines are sigmoidal curve fits.

We next analyzed the kinetics of PR activation and substrate cleavage in nascent virions using two parameters derived from the nFRET traces (Figure 1D and Figure 3A,B). First, we measured the waiting time to PR activation (T_PR_), defined as the interval from the appearance of a YFP/mCherry punctum detectable above background to the onset of nFRET reduction (Figure 3B, green-shaded segment). T_PR_ likely includes the time required for pseudoviral assembly, budding, and PR activation leading to detectable cleavage of the FRET substrate. Second, we measured the substrate cleavage time (ΔT), defined as the time from PR activation to completion of substrate cleavage, defined as the transition time from high to low nFRET (Figure 3B, pink-shaded segment). ΔT likely reflects the time required for complete proteolysis of a PR substrate in single virions. Both kinetic parameters were obtained by curve-fitting nFRET traces with a sigmoidal function (Figure 3B, dashed line). Here, the onset of substrate cleavage and completion of substrate cleavage were defined as 10% and 90% drops in nFRET signal, respectively (Figure 3B).

It should be stressed that the two parameters—waiting time for protease activation (T_PR_) and substrate cleavage time (ΔT)—do not correlate (Supplementary Figure S6A). Thus, the interval from particle appearance to PR activation is not a predictor of the rate of substrate cleavage (i.e., early activation does not lead to faster or slower substrate processing). Analysis of PR activation and substrate cleavage in nascent particles was performed by plotting the cumulative fractions of events against time (Figure 3C,D). Interestingly, the observed distributions exhibit an apparent sigmoidal shape with short lag times. This indicates the existence of multiple sequential steps preceding a detectable drop in nFRET and transition to a low nFRET value due to the completion of substrate cleavage by HIV-1 PR. Overall, PR activation occurred with a half-time of 18.5 min after the onset of viral assembly, while the FRET substrate cleavage after PR activation reached completion with a half-time of 2.2 min. 

We next investigated whether ΔT is proportional to the number of substrate molecules per virion (assessed by measuring the integral mCherry signal per particle). The lack of correlation between ΔT and the integral mCherry intensity per particle (Supplementary Figure S6B) indicates that the amount of FRET substrate is not rate-limiting and that instead, the cleavage time is largely determined by the varied PR activity in individual particles. Accordingly, the apparent substrate processing times were not considerably affected by altering the ratio of mCherry-2xcl-YFP-Vpr and the HIV-1 backbone plasmids that may modulate the amount of substrate per virion (Supplementary Figure S7).

### 2.4. A Larger Fraction of Cell-Free Virions Contains Cleaved nFRET Substrate Compared with Particles Assembled on the Plasma Membrane

**Figure 4:**
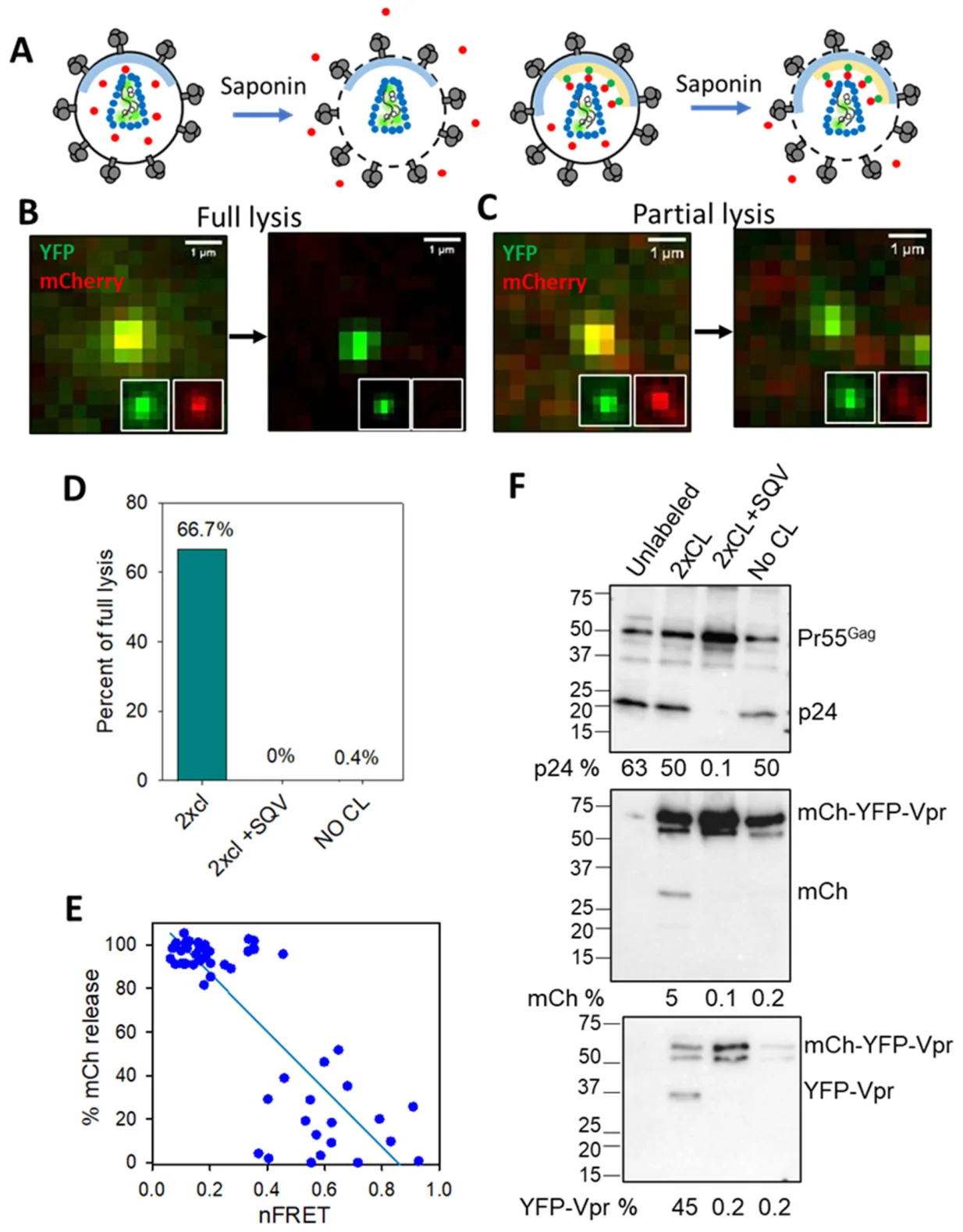
FRET substrate cleavage in cell-free virions. (**A**) A cartoon of virus lysis with saponin resulting in full or partial release of free mCherry (red dots) produced by cleavage of mCherry-2xcl-YFP-Vpr by PR (YFP is shown by green dots). (**B**,**C**) Images of saponin-mediated lysis of 293T cell-produced pseudoviruses attached to a coverslip. Examples of full (**B**) and partial (**C**) release of mCherry upon application of 0.1 mg/mL saponin are shown. (**D**) Analysis of mCherry release from single virions exposed to saponin for a representative viral panel consisting of mCherry-2xcl-YFP-Vpr (with or without SQV pretreatment) and virions containing the uncleavable mCherry-YFP-Vpr substrate (NO CL). (**E**) Correlation between nFRET values and the extent of mCherry release from single virions containing the mCherry-2xcl-YFP-Vpr substrate upon exposure to saponin. Solid line is linear regression (R^2^ = 0.70). (**F**) Immunoblot analyses of mCherry-2xcl-YFP-Vpr viruses from panels D and E. Densitometry results for the extent of Gag or substrate cleavage are included below the respective blots.

**Figure 5:**
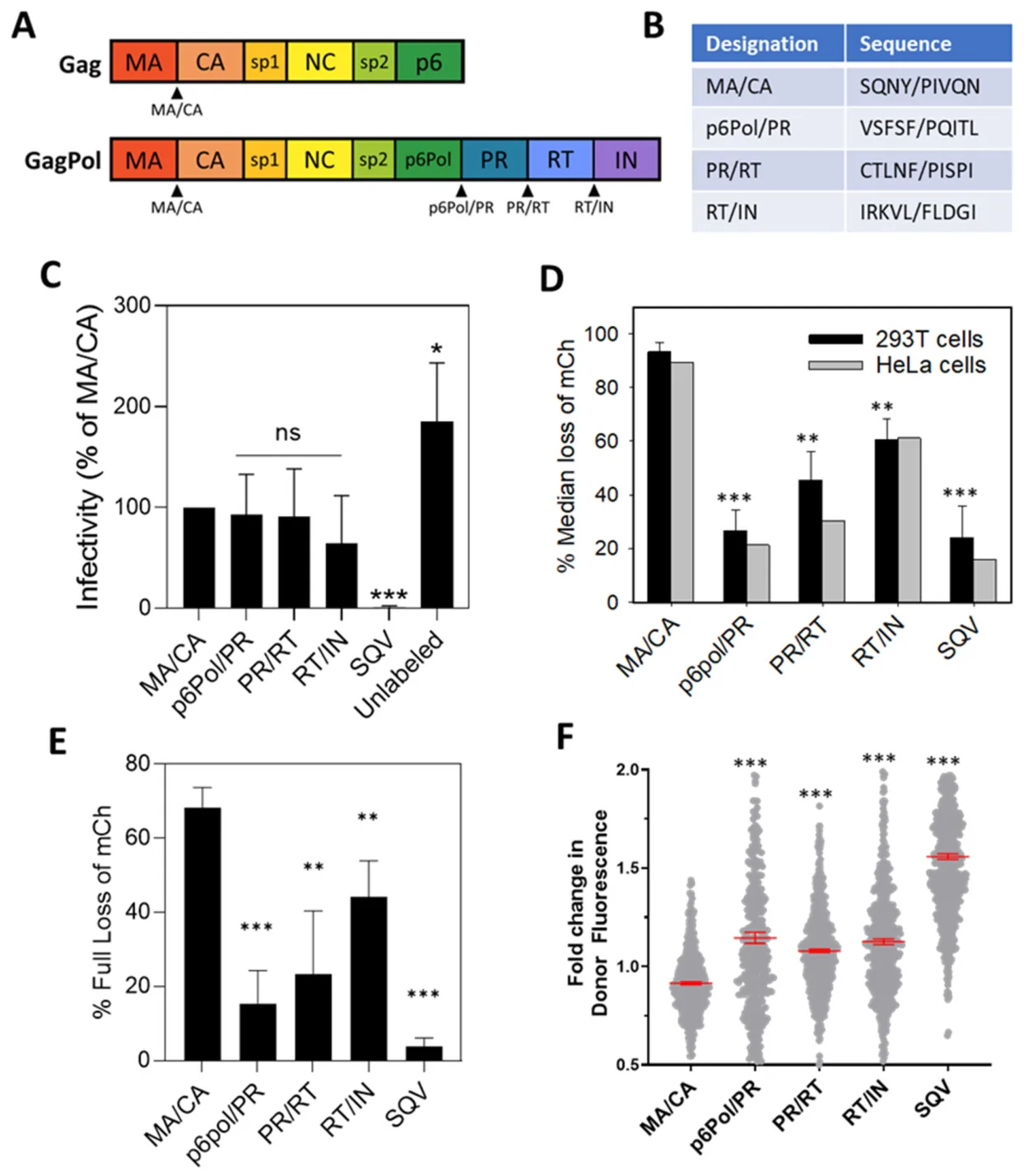
The extent of surrogate substrate cleavage by PR is sequence-dependent. (**A**) Schematic of Gag and Gag-Pol polyproteins, with arrows indicating the HIV-1 protease cleavage sites cloned into the Vpr-based FRET sensor. (**B**) Table of selected PR cleavage sequences across MA/CA, RT/IN, p6Pol/PR, and PR/RT domains. (**C**) Infectivity of pseudoviruses containing FRET substrates with different PR cleavage sites and produced by transfection of 293T cells. Equal amounts of p24 of different pseudoviruses were used to infect TZM-bl cells, and the resulting luciferase signal was measured at 48 hpi. Results for four independent pseudovirus preparations are shown. (**D**) The extent of mCherry release (median fraction of mCherry loss) upon lysis with 0.1 mg/mL saponin of a panel of pseudoviruses containing distinct PR substrates produced in 293T cells (8 independent experiments) or HeLa cells (a representative experiment is shown). The measurable release of mCherry from SQV-treated virions may be due to incomplete inhibition of the PR precursor by this compound. (**E**) The extent of mCherry release plotted as the fraction of particles exhibiting “full” loss of mCherry upon saponin lysis (see Supplementary Figure S9). (**F**) The FRET efficiency for substrates with different PR cleavage sites in cell-free virions adhered to coverslips was assessed by photobleaching mCherry and measuring the resulting increase in donor (YFP) signal. Horizontal red line and error bars represent mean fold-increase and standard error, respectively. In panels C-F, SQV indicates MA/CA substrate-containing virions produced in the presence of 500 nM SQV. Statistical analysis was performed using a Mann–Whitney test. ns, not significant; *, *p* < 0.05; **, *p* < 0.01; ***, *p* < 0.001.

We sought to compare the fraction of virions undergoing reduction in nFRET during live-cell imaging with the extent of mCherry-2xcl-YFP-Vpr substrate in virions released by transfected 293T cells. The extent of substrate processing was assessed using two orthogonal approaches. First, we measured the fraction of mCherry release from coverslip-adhered pseudoviruses after permeabilizing the viral membrane with saponin (Figure 4A). For virions in which the FRET substrate was fully cleaved, permeabilization led to release of all mCherry molecules, leaving no residual signal above background (Figure 4B, “Full lysis”). However, a fraction of virions retained detectable levels of mCherry after lysis, consistent with incomplete cleavage of the Vpr-based FRET substrate in virions (Figure 5C, “Partial lysis”). Approximately 67% of particles released a portion of mCherry that exceeded a set threshold (>70%, termed “full lysis”) upon exposure to saponin (Figure 4D). This threshold was determined based upon the distributions of mCherry loss from control particles produced in the presence of SQV or containing a FRET substrate lacking the PR cleavage site (Supplementary Figure S8). As expected, higher nFRET values for these control viruses correlated with the markedly lower fraction of mCherry released after saponin lysis (Figure 4E and Figure S8).

We next assessed the extent of Gag and substrate cleavage by Western blotting. The incorporation of mCherry-2xcl-YFP-Vpr or mCherry-YFP-Vpr (a FRET sensor without a cleavable linker between the acceptor and donor) did not significantly affect the efficiency of Gag processing, whereas SQV treatment blocked Gag cleavage, as expected (Figure 4F, top). In comparison, the blotting of viral lysates for mCherry showed the major mCherry-2xcl-YFP-Vpr precursor band and a minor band (5%; Figure 4F, middle) corresponding to free mCherry. Interestingly, blotting for GFP/YFP revealed a more prominent band corresponding to the YFP-Vpr cleavage product relative to the precursor (45%; Figure 4F, bottom), which is more in line with the saponin lysis results (Figure 4D). The discrepancy between the relative extents of substrate cleavage in mCherry and GFP/YFP blots may be related to the lower efficiency of mCherry transfer compared with the mCherry-2xcl-YFP-Vpr precursor. As expected, these cleavage products were abrogated by SQV treatment or when using the uncleavable FRET substrate. Collectively, our results imply that a larger fraction of released virions contain the fully or partially cleaved FRET substrate compared with a minor fraction of virions assembling on the ventral membrane of HeLa cells that exhibit reduction in nFRET (Figure 2E).

### 2.5. FRET Substrates Containing Alternative PR Cleavage Sequences Are Processed with Different Efficiency 

It has been shown that the cleavage rates of a surrogate MA-CA-based substrate by recombinant PR in vitro rank as follows: MA/CA > RT/IN > SP1/NC ≥ CA/SP1 > TF/PR ≥ PR/RT ≥ SP2/p6 > NC/SP2 [11]. To test whether the PR cleavage sequence affects the kinetic and extent of FRET substrate processing in single virions, the chimeric 2xcl site in the mCherry-2xcl-YFP-Vpr construct was replaced with distinct protease cleavage sequences linking different Gag and Gag-Pol domains (Figure 5A). We focused on four cleavage sequences—one within Gag (MA/CA) and three within Pol (p6Pol/PR, PR/RT, and RT/IN; Figure 5B). The MA/CA and RT/IN cleavage sites were selected because these have been reported to support the highest cleavage rates by PR in vitro, whereas the p6Pol/PR and PR/RT cleavage rates were among the lowest [11].

The PR cleavage sequence in our FRET substrate did not significantly alter virions’ specific infectivity measured using a single-cycle luciferase reporter assay, but viruses containing these Vpr-based substrates were ~2-fold less infectious compared with control viruses lacking the substrate (Figure 5C). These results imply that the efficiency of Gag processing and specific infectivity are insensitive to the PR cleavage sequences inserted into our FRET reporter. We note, however, a considerable preparation-to-preparation variability in specific infectivity of independent virus panels resulting in large error bars.

To determine if the PR cleavage sequence impacts the extent of FRET substrate processing, we measured the fraction of mCherry released from cell-free virions permeabilized with saponin. Tracking single-particle fluorescence intensities containing distinct PR cleavage sequences revealed both full and partial mCherry release events after saponin application, albeit their relative fractions differed markedly (Supplementary Figure S9A–E). Particles with the MA/CA and RT/IN substrates exhibited bimodal distributions of lysis efficiency, with low and high fractions of released mCherry corresponding to uncleaved and fully cleaved substrates, respectively. By contrast, most p6Pol/PR-containing particles released only a fraction of mCherry, suggesting the poor processing of this substrate in pseudoviruses. PR/RT-containing particles exhibited an intermediate lysis phenotype, with a lager fraction of pseudoviruses releasing the entire mCherry pool than those containing the p6Pol/PR substrate.

Loss of mCherry from saponin-treated single virions was parameterized by calculating the percent of particles releasing an above-threshold fraction of mCherry (“full” release) or using the median fraction of released mCherry determined from single-particle lysis data. Analyses of different FRET substrates across several independent viral panels revealed a significantly more efficient processing of MA/CA compared with the rest of PR substrates, with RT/IN also being processed relatively efficiently (Figure 5D,E). Comparison of pseudoviruses produced in 293T and HeLa cells implies that the efficiency of FRET substrate cleavage by PR is independent of producer cells (Figure 5D). Alternatively, we parameterized mCherry loss by plotting the ratio of YFP+ over mCherry+ particles as a function of time after saponin addition (Supplementary Figure S10). This analysis further highlights the superior processing of MA/CA relative to other PR cleavage sequences. For a panel of PR substrates, the extent of mCherry release from virions inversely correlated with the FRET efficiency determined by acceptor photobleaching (Figure 5F). For instance, the lowest FRET values for MA/CA particles were associated with the most complete mCherry release upon virus lysis. Overall, loss of mCherry from virions ranked as follows: MA/CA > RT/IN ≥ PR/RT > p6Pol/PR (Figure 5D,E).

To exclude the possibility that the residual fraction of mCherry after saponin lysis is due to sequestration of this marker within intact HIV-1 capsids, control experiments were performed in the presence of 20 µM PF74. At this concentration, PF74 is known to hyper-stabilize the HIV-1 capsid and induce structural defects that allow for the release of encapsulated fluorescent proteins (e.g., [40,41,42]). However, PF74 treatment had no effect on the fraction of unreleased mCherry remaining after saponin lysis (Supplementary Figure S11), indicating that saponin-released mCherry accurately reports the fraction of the mCherry-cl-YFP-Vpr substrate processed by PR, without significant retention of proteolytically processed mCherry within intact viral cores.

**Figure 6:**
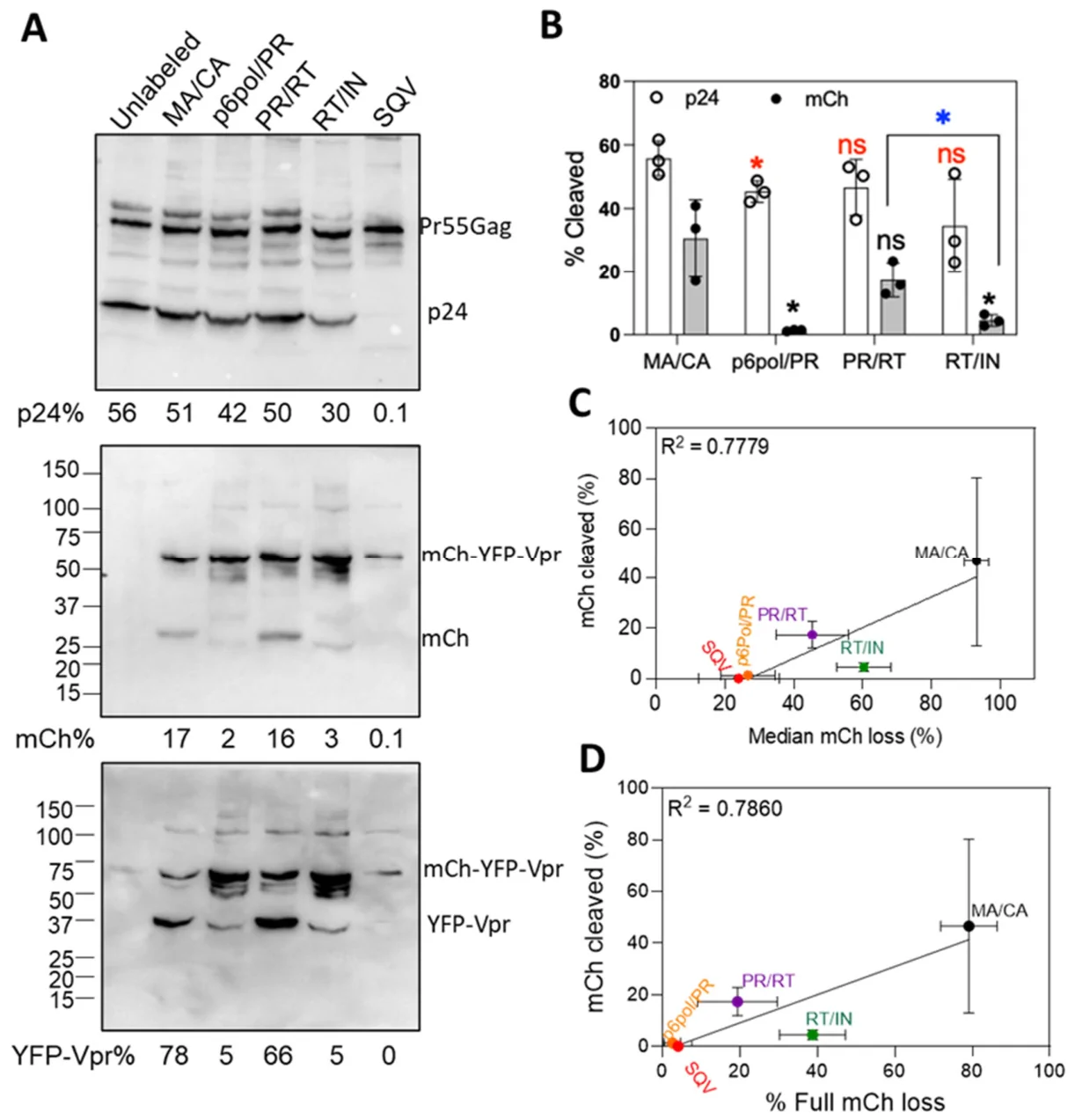
Immunoblot analyses of Gag and FRET substrate cleavage. (**A**) Western blotting results for a representative panel of pseudoviruses bearing distinct PR cleavage sites inserted into the Vpr-based FRET substrate. Pseudoviruses containing different FRET substrates were lysed, subjected to SDS-PAGE and blotted with anti-HIV immune serum (top panel), anti-mCherry antibody (middle panel), or anti-GFP antibody (lower panel). The fractions of cleaved Gag, mCherry and YFP-Vpr determined by densitometry are shown below the blots. (**B**) The extents of Gag and FRET substrate cleavage for four independent virus preparations are plotted. Statistical analysis was done using a Mann–Whitney test. *, *p* < 0.05. Red asterisks show significance level for Gag cleavage, whereas the blue asterisk indicate the level of significance between mCherry processing between PR/RT and RT/IN. (**C**,**D**) Correlations between substrate processing determined by Western blotting and percent of particles releasing a median fraction of mCherry (**C**) and losing nearly all mCherry (>70%) signal (**D**). In panels A, C and D, SQV indicates MA/CA substrate-containing virions produced in the presence of 500 nM SQV. The extents of substrate cleavage from 4 independent preparations are replotted from Figure 5D,E. Lines are the linear regressions.

We next examined Gag and FRET substrate processing in virions by SDS-PAGE and Western blotting for p24 and mCherry, respectively (Figure 6A,B). Across several viral preparations, the extent of Gag processing (p24/Gag Pr55 band ratio) was not significantly modulated by the cleavage sequence within the FRET substrates (Figure 6A,B and Supplementary Figure S13), as expected. The processing of Gag precursor in virions lacking the Vpr-based FRET sensor (“Unlabeled”) was more efficient than in those containing the FRET substrate (Figure 6A and Figure S12), in line with the higher specific infectivity of the former virions (Figure 5C). Compared with Gag processing, the mCherry blots revealed marked variations in the efficiency of mCherry-cl-YFP-Vpr cleavage. Whereas robust free mCherry bands were detected for MA/CA and PR/RT substrates, p6Pol/PR and RT/IN substrates were less efficiently processed (Figure 6A,B and Figure S12). As with the 2xcl substrate cleavage (Figure 4F), blotting for GFP/YFP yielded a more prominent cleavage product, thus indicating a higher apparent cleavage efficiency compared with the mCherry blot (78% vs. 17%, respectively; Figure 6A). Of note, the relative substrate processing efficiency values determined by immunoblotting for mCherry and based upon the extent of mCherry loss upon saponin lysis correlated (R^2^ > 0.77; Figure 6C,D). Collectively, our results imply that the PR cleavage sequences from Gag and Gag-Pol polyproteins linking our donor/acceptor FRET pair are processed with different efficiency in virions.

### 2.6. The Rate of FRET Substrate Processing Is Modulated by the PR Cleavage Sequence

**Figure 7:**
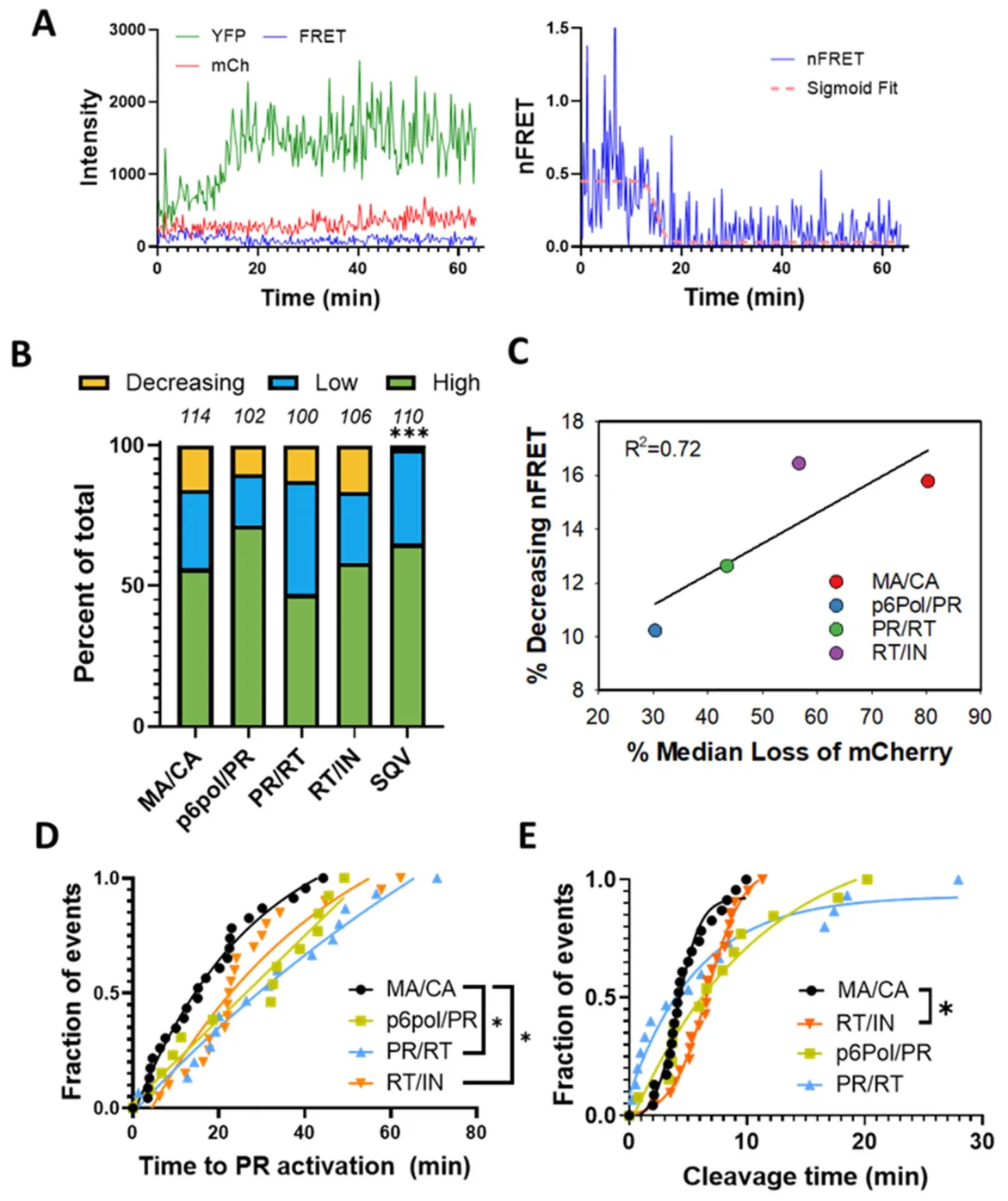
The rate and extent of FRET substrate processing are modulated by PR cleavage sequence. (**A**) Representative YFP and mCherry fluorescence intensity tracks (left) and nFRET trace (right) showing PR activity in a single particle containing the MA/CA substrate. (**B**) Categorization of the nFRET values for the newly forming particles containing distinct PR cleavage sites, with decreasing nFRET values indicating PR activation in nascent virions. Randomly selected nascent puncta were analyzed, and their nFRET values were categorized into high (>0.25), low (<0.25), and decreasing. SQV stands for mCherry-2xCl-YFP-Vpr-containing particles imaged in the presence of 500 nM SQV (replotted from Figure 2E). The numbers of analyzed particles are given above the graph. (**C**) Correlation between the fraction of assembling virions exhibiting reduction in nFRET (from panel (**B**)) and the extent of mCherry release from cell-free virions lysed by saponin (from Figure 5D). (**D**) Distributions of waiting times for protease activation for MA/CA, RT/IN, p6Pol/PR, and PR/RT cleavage sites. Solid lines are exponential curve fits with R^2^ > 0.96 for all but RT/IN (R^2^ > 0.90) substrates. (**E**) Distributions of substrate cleavage times for different FRET substrates measured as the transition times from high to low nFRET (see Section 4). Solid lines are exponential curve fits for p6Pol/PR and PR/RT (R^2^ = 0.94 for both), and sigmoidal curve fits for MA/CA and RT/IN (R^2^ = 0.98 and 0.99, respectively). Statistical analysis was performed using a Fisher’s Exact test for panel (**B**), and a Mann–Whitney test for panels (**D**,**E**). *, *p* < 0.05; ***, *p* < 0.001.

The PR activation events in nascent particles assembled at the ventral membrane were imaged by TIRFM. An example of MA/CA substrate cleavage event is shown in Figure 7A. Analysis of *de novo* forming particles with different PR cleavage sequences in our Vpr-based FRET substrate revealed a modest variation in the fraction of nascent virions with stable/high or stable/low nFRET signals and those with decreasing nFRET signal (Figure 7B). The fraction of assembled p6Pol/PR particles exhibiting a decrease in nFRET signal was lower than for other constructs, although this difference did not reach statistical significance. The extent of mCherry release for different PR substrates (from Figure 5D) correlated with the fraction of nFRET decrease events in live-cell experiments (Figure 7B,C). This correlation supports a link between early PR activation at the ventral membrane and the overall cleavage efficiency of FRET substrates in the entire population of released virions.

We next examined the dependence of PR activation kinetics (T_PR_) on the cleavage sequences within our FRET substrate. In virions containing the MA/CA cleavage site, PR activation occurred significantly faster than in those containing the PR/RT or RT/IN cleavage sequences (Figure 7D). The p6Pol/PR kinetic was also slower than that for MA/CA, but the difference was not significant. These findings are somewhat surprising, since the PR cleavage sequence inserted within a surrogate Vpr-based substrate should not modulate the timing of PR activation. The differences in T_PR_ distributions may be caused, in part, by variations in signal-to-background ratio for distinct FRET substrates, which would affect the ability to detect the onset of particle assembly and the timing of initial nFRET decrease that define T_PR_ (illustrated in Figure 3B). 

Analysis of FRET substrate processing revealed that the cleavage times were modulated by the sequences linking mCherry and YFP (Figure 7E). The cleavage times for the PR-flanking sequences (p6Pol/PR and PR/RT) were exponentially distributed (Figure 7E; R^2^ = 0.94 for both constructs), suggesting a first-order reaction. Note that the cleavage times of p6Pol/PR and PR/RT were distributed over a broad range. Examples of quick (~1 min) and slow (>15 min) PR/RT substrate processing are shown in Supplementary Figures S13 and S14, respectively. By comparison, the distributions of cleavage times of MA/CA and RT/IN deviated from the exponential distribution (R^2^ is 0.78 and 0.69, respectively) and could be fitted with a sigmoidal function (Figure 7E). Although the cleavage times tended to vary, only the PR/RT cleavage times were statistically different from MA/CA and RT/IN, perhaps reflecting a limited sample size for these data sets. The PR/RT cleavage time was borderline faster than that of p6Pol/PR (*p* = 0.055; Figure 7E). We note that the somewhat slower rate of p6Pol/PR processing tracks with its poor cleavage efficiency (Figure 5 and Figure 6). The median substrate processing times ranked as follows: MA/CA = PR/RT (4.1 min) < p6Pol/PR (6.2 min) < RT/IN (6.6 min).

## 3. Discussion

Our results demonstrate that the HIV-1 PR activity can be monitored in nascent virions based upon reduction in FRET signal from a Vpr-based substrate. FRET-based protease substrates are broadly used in cell biology and virology, but our FRET probe enables the real-time monitoring of PR activity in single assembling virions. Loss of nFRET was detected in a relatively small fraction of assembled particles (~13%) within our imaging window (Figure 2E and Figure 7B). The rarity of detected PR activation events is consistent with a previous work that employed single-virus tracking and fluorescence lifetime imaging to detect loss of FRET due to protease activity in nascent virions [29]. In fact, that study detected a drop in reporter fluorophore lifetime in only 6% of virions assembled on the ventral membrane.

In contrast to the relatively low probability of PR activation in nascent particles assembling on the ventral membrane, most cell-free 2xcl or MA/CA virions contain a fully cleaved FRET substrate, as evidenced by immunoblot analysis and saponin lysis results (Figure 4 and Figure 6). This suggests that in most virions assembling on the ventral membrane, PR activation may be delayed relative to our imaging window of 2 h, which is limited by the crowding of assembled virions on the ventral membrane. Alternative possibilities include (1) insufficient sensitivity of our FRET-based assay to detect partial substrate cleavage events and (2) more efficient PR activation in virions assembled on the dorsal membrane, which cannot be imaged by TIRF microscopy. Indeed, HIV-1 assembly/release from the cell’s dorsal membrane imaged by other techniques has been reported to occur more quickly than on the ventral membrane [43]. Since the PR precursor has been shown to relatively efficiently cleave a FRET substrate [27], it is also possible that a slower substrate cleavage by the precursor may escape detection in live-cell imaging experiments but manifest itself in the released virions. This would explain the much higher fraction of virions containing a fully cleaved substrate in cell-free vs. assembling particles, as assessed by saponin lysis and nFRET drop experiments, respectively. 

Surprisingly, a fraction of HIV-1 particles exhibited a low initial nFRET signal at the time of assembly (Figure 2E and Figure 7B). This may suggest early PR activation before virion budding. However, if PR activation occurs in the cytoplasm, the assembling particles should exhibit a diminished mCherry signal relative to the YFP signal. Similarly, if such early cleavage events occur during viral assembly, loss of mCherry from fluorescent spots to the cytosol should be detected, unless the neck of a budding virion is too constricted to allow for mCherry diffusion. Neither of these events were observed in our experiments (e.g., Figure 1B,E). In addition, low-nFRET particles were seen in the presence of SQV (Figure 2E and Figure 7B). It also seems unlikely that low initial nFRET is caused merely by a delayed detection of assembled particles against the high local background that masks PR activation and substrate cleavage events. Thus, the nature of these low-nFRET particles remains unclear at present.

The tracking of single-particle assembly, combined with nFRET measurements, enabled kinetic analysis of PR activation and substrate processing, using two parameters—time between appearance of a fluorescent Vpr punctum and onset of substrate cleavage (T_PR_) and the nominal time required for substrate processing (ΔT). T_PR_ includes the steps of viral assembly, budding and the lag to PR activation (if any) and, thus, is not a direct measure of the timing of PR activation. Fidelity of T_PR_ measurements depends on the ability to detect the onset of viral assembly against variable background and the time required for significant substrate cleavage by PR (initial drop in nFRET). The half-time for PR activation in our experiments was ~20 min (Figure 3C and Figure 7D), in line with the previously reported 25–30 min time interval from the onset of assembly to virus release [44,45]. Based on the reported HIV-1 assembly times (without release), which vary between 3 and 9 min [29,44,45], particle assembly should majorly contribute to T_PR_. Our results are also in general agreement with a 14 min delay between onset of assembly and cleavage of the Gag-iCFP reporter monitored by fluorescence lifetime microscopy [29]. Given the half-time of assembly ~3 min in that study, PR activation may occur within ~11 min post-assembly—a lag that likely includes the time for virus budding. However, since we did not directly monitor virus budding, the possibility that PR activation occurs at the time or within a few seconds after HIV-1 release cannot be ruled out [27,28].

In our experiments, cleavage of a FRET substrate by PR in single virions was completed within a few minutes (Figure 3D and Figure 7E). This timing is much shorter than the T_50_ of ~20 min for Gag cleavage using bulk measurements of PR activation in released virions after a protease inhibitor washout [17]. Likewise, PR inhibitor photoinactivation and single-virus STED imaging yielded a T_50_ of 29 min for core condensation after Gag processing [18]. These differences could reflect the differential accessibility of a Vpr-based substrate vs. Gag polyprotein and/or be due to suboptimal/aberrant PR activity after removal of an inhibitor. However, we cannot rule out the possibility that our FRET assay is biased toward particles in which PR activation occurred early.

We found that the cleavage time distributions for the PR-flanking p6Pol/PR and PR/RT sequences could be fitted with an exponential function, whereas MA/CA and RT/IN cleavage times were better fitted with a sigmoidal function (Figure 7E). A complex kinetic of FRET substrate cleavage in virions is expected, as the initial cleavage may be executed by the PR precursor, while the formation of mature dimeric PR over time could accelerate the rate of cleavage. MA/CA processing was significantly faster than PR/RT cleavage. The lack of significant differences between MA/CA and the rest of PR cleavage sites might be due to limited sample sizes. A somewhat slower cleavage of p6Pol/PR in live-cell experiments and its inefficient processing in cell-free virions compared with MA/CA and other cleavage sequences (Figure 5D,E and Figure 7E) suggests that this cleavage event may be rate-limiting. With the caveat of comparing the exponential vs. sigmoid cleavage kinetics, our substrate cleavage times rank as: MA/CA = PR/RT < p6Pol/PR < RT/IN. We note that the distributions of waiting time for PR activation and the substrate cleavage times for MA/CA were close to those for 2xcl, which contains the MA/CA sequence, suggesting that the chimeric 2xcl sequence does not majorly impact the PR cleavage rate or efficiency.

The in vitro cleavage rates by recombinant PR of an artificial PR substrate consisting of the MA and CA domains linked by distinct cleavage sequences could be fitted with an exponential function and ranked as follows: MA/CA > RT/IN > p6Pol/PR ≥ PR/RT [11]. Also, the faster processing of MA/CA and RT/IN sites within Gag-Pol by recombinant PR relative to the PR-flanking cleavage sites (p6Pol/PR and PR/RT) has been reported [12]. The differences in the relative cleavage rates in our experiments and in vitro experiments stress the importance of studying PR activity in the context of intact virions.

In parallel with the kinetic measurements of nFRET reduction, we assessed the efficiency of substrate cleavage by: (1) measuring nFRET distributions for single virions, (2) analyzing viral lysates by Western blotting for mCherry and YFP-Vpr, and (3) measuring the fraction of mCherry released from virions upon lysis with saponin. The key observation from these experiments is that the extent of FRET substrate processing is modulated by the PR cleavage sequence. Saponin lysis data revealed a large fraction of virions containing partially cleaved pools of p6Pol/PR and PR/RT substrates (Supplementary Figure S9). The results of immunoblot analysis of substrate processing are in general agreement with the extent of mCherry release upon saponin lysis (Figure 6C,D). Our results agree with the more efficient cleavage of MA/CA and RT/IN by mature PR in vitro compared with cleavage of p6Pol/PR (TF/PR) and PR/RT [12].

The reasons for the partial processing of Vpr-based PR substrates in HIV-1 particles observed in this study are not understood. Given that MA/CA-derived mCherry is rather efficiently released by saponin treatment and based upon immunoblot analysis, the poor processing of other substrates is unlikely due to a limited access of PR in immature particles. It is possible that the slower cleavage of these substrates occurs on the time scale of PR inactivation, thus leaving unprocessed pools of these substrates in virions.

The relatively invariable fraction of assembling virions exhibiting reduction in nFRET (Figure 7B) and the same magnitude of nFRET drop (Supplementary Figure S15) across the PR cleavage sequences tested contrast with the marked differences in the substrate processing efficiency in cell-free virions (e.g., Figure 5 and Figure 6). Nonetheless, the correlation between the fraction of mCherry release from lysed virions and the fraction of nascent particles exhibiting loss of nFRET in live-cell experiments (Figure 7C) indicates a potential link between early activation of PR and completion of substrate cleavage in released virions. Future studies of additional PR cleavage sequences from HIV-1 Gag using our FRET-based assay will help delineate the timing of PR activation, along with the intrinsic rates and efficiency of substrate processing in single virions.

## 4. Materials and Methods

### 4.1. Cell Lines, Reagents, and Plasmids

HeLa and HEK293T/17 cells obtained from ATCC (Manassas, VA, USA) were grown in Dulbecco’s Modified Eagle Medium (DMEM) supplemented with 10% FBS and 1% penicillin–streptomycin (Gemini Bio-Products, Sacramento, CA, USA). Medium for HEK293T/17 cells was additionally supplemented with 0.5 mg/mL G418 (Cellgro, Mediatech, Manassas, VA, USA). Heat-inactivated FBS and poly-L-lysine were from Sigma-Aldrich (St. Louis, MO, USA). The following reagents were obtained from the NIH HIV Reagent Program/BEI Resources: saquinavir (contributed by DAIDS/NIAID) and human anti-HIV serum (NIH HIV Reagent Program Cat# 3957, RRID:AB_2890264); Human Immunodeficiency Virus 1 (HIV-1) HXB2 Rev expression vector (pcRev) was contributed by Dr. Bryan R. Cullen.

Goat anti-human from Bio-Rad (Hercules, CA, USA), HRP-conjugated mouse anti-rabbit from Sigma (Burlington, MA, USA), rabbit anti-mouse-HRP (Southern Biotech, Birmingham, AL, USA), rabbit anti-mCherry from Invitrogen (Carlsbad, CA, USA), and mouse-anti GFP JL-8 (Takara Bio, San Diego, CA, USA) were used for Western blotting. TransIT-HeLaMONSTER transfection reagent (Mirus Bio LLC, Madison, WI, USA) was used for transfection of HeLa cells, while JetPrime transfection reagent and JetPrime buffer (Polyplus-transfection, SA, New York, NY, USA) were used for HEK293T/17 cells.

The pCAGGS plasmid encoding HXB2 Env was provided by Dr. J. Binley (Torrey Pines Institute). The pR9ΔEnvΔNef vector was provided by Dr. Christopher Aiken (Vanderbilt University). DNA encoding for the protease cleavage sites (PCS) of HIV-1 Gag and Gag-Pol domains (MA/CA: SQNY/PIVQN; p6/PR:VSFSF/PQITL; PR/RT: CTLNF/PISPI; and RT/IN: IRKVL/FLDGI) was PCR-amplified using primers with overhangs that contained the PCS sequence in frame with the N-terminus of YFP as PCS-YFP DNA fragments containing flanking XhoI and BamHI restriction enzyme sites. The DNA encoding for mCherry was amplified from HIV-1 Gag-imCherry with flanking HindIII and XhoI restriction enzyme sites. The two DNA fragments were later digested with HindIII + XhoI (mCherry) and XhoI + BamHI (PCS-eYFP), cloned into pMM310-mCherry-2xCL-YFP-Vpr [36] by excising mCherry-2xCL-YFP using enzymes HindIII and BamHI and by three-way ligation of DNA fragments, and sequence-verified by whole plasmid sequencing (Plasmidsaurus, San Francisco, CA, USA).

### 4.2. Cell Transfection and Pseudovirus Production

HEK293T/17 cells (70% confluent, 10 cm dish) were transfected with JetPrime transfection reagent and JetPrime buffer and the following plasmids: 3 µg of HXB2 Env, 4 µg of pR9ΔEnvΔNef, 1 µg of Rev, and 2 µg of mCherry-cl-YFP-Vpr with different PR cleavage sites (or mCherry-2xcl-YFP-Vpr construct [36]), amounting to 10 µg of plasmid per dish. For Western blot analysis, the same transfection procedure was used, except 1 µg of mCherry-cl-YFP-Vpr was used instead of 2. Culture medium was replaced with fresh medium 18 h post-transfection (hpt), either with or without 500 nM SQV, and the supernatant was collected at 48 hpt. The supernatant was filtered through a 0.45 µm filter, concentrated by LentiX (Clontech, Mountain View, CA, USA), spun down at 1500 × rcf for 45 min, aliquoted, and stored at −80 °C until use. p24 content in viral stock was determined by ELISA, as described in [46].

HeLa cells were seeded on collagen-coated glass-bottom 35 mm dishes (No. 1.5, MatTek, Ashland, MA, USA) and transfected with 0.6 µg of HXB2, 0.8 µg of pR9ΔEnvΔNef, 0.4 µg of mCherry-cl-YFP-Vpr, and 0.2 of µg pcRev (a total of 2 µg of plasmid per 35mm dish) using TransIT-HeLaMONSTER. At 18 hpt, the transfection mixture was replaced with fresh growth medium.

### 4.3. Western Blotting

Laemmli SDS-PAGE loading buffer (Bio-Rad) and β-mercaptoethanol (Sigma-Aldrich) were added to the virus samples, and the mixture was heated to 95 °C for 5 min. A total of 30 pg of p24 was loaded and ran on 4–15% precast polyacrylamide gel (Bio-Rad, Hercules, CA, USA). Precision Plus Protein Standards (Kaleidoscope, Bio-Rad) was used as a molecular weight marker. Proteins separated by SDS-PAGE were semi-dry-transferred to a nitrocellulose membrane and blocked with 10% blotting-grade blocker (BioRad, Hercules, CA, USA) in PBST (PBS + 0.1% Tween) for 30 min at room temperature. Membranes were then incubated overnight with human anti-HIV serum (1:2000 dilution) or rabbit anti-mCherry (1:500 dilution) or mouse anti-GFP (JL-8) (1:500 dilution). After 3 × 10 min washes with PBST, membranes were incubated for 1 h at room temperature with HRP-conjugated goat anti-human (1:2000), HRP-conjugated mouse anti-rabbit (1:2000 dilution) or rabbit anti-mouse HRP-conjugated (1:2000 dilution). Membranes were imaged on a Gel Doc imager (Bio-Rad), and bands were analyzed using ImageLab (Bio-Rad, Version 3.0).

### 4.4. Single-Virus Lysis

Virus was attached to a poly-L-lysine-coated 8-well chamber coverslip (Lab-Tek, Nalge Nunc International, Penfield, NY, USA) for 30 min at room temperature, washed with PBS, and imaged on a DeltaVision Elite microscope using a TIRFM PlanApoN 60x/1.45NA oil objective (Olympus, Tokyo, Japan) and a GFP/mCherry live-cell filter set. Samples were imaged at a rate of 6 s/frame for 4 min. To permeabilize the viral membrane and release the viral content marker, mCherry, saponin was added after 10 frames at a final concentration of 0.1 mg/mL. The acquired movies were used to track single viral particles using ICY software (icy.bioimageanalysis.org, last accessed on 4 May 2026) and analyzed in Excel. Only particles with mCherry signal exceeding 1000 a.u. before lysis were included in the analysis. From the obtained tracks, the extent of mCherry release upon lysis was parameterized by: (1) fraction of particles exhibiting “full loss” of mCherry (defined as loss of at least 70% of mCherry intensity after saponin addition and (2) fraction of particles exhibiting median loss of mCherry intensity. Alternatively, the extent of mCherry release was quantified by a fold change in the ratio of YFP-positive (green) over mCherry-positive (red) particles after lysis, using the ComDet ImageJ plugin (version 1.54p).

### 4.5. Live-Cell Imaging

Transfected HeLa cells were imaged 43–47 hpt on a DeltaVision Elite microscope (Leica Microsystems, Wetzlar, Germany) using a TIRF PlanApoN 60x/1.45NA oil immersion objective (Olympus, Tokyo, Japan) and a GFP/mCherry filter set, in a humidity-controlled chamber at 37 °C. Prior to imaging, the growth medium was replaced with live-cell imaging buffer (Life Technologies, Inc., Grand Island, NY, USA) supplemented with 10% FBS. 

### 4.6. TIRF Alignment and Normalized FRET (nFRET) Calculation

To align TIRF illumination, 0.1 µm Tetraspeck microspheres (Invitrogen) were sonicated for 5 min, diluted 1:200 in PBS, and attached to a poly-L-lysine-coated 8-well chamber coverslip (Lab-Tek, Nalge Nunc International, Penfield, NY, USA) for 30 min at room temperature. After washing with PBS to remove unattached beads, 80 nM fluorescein solution was added. The sample was then imaged using a GFP/mCherry live-cell filter set and a TIRF PlanApoN 60x/1.45NA oil immersion objective (Olympus, Tokyo, Japan) on a DeltaVision Elite microscope (Leica Microsystems, Wetzlar, Germany). The TIRF illumination incident angle was then decreased until the background fluorescence from fluorescein was suppressed, while Tetraspeck beads remained clearly visible across the image field. This setting was used for TIRF imaging of viruses and live cells. 

When collecting mCherry emission upon 488 nm excitation (sensitized emission), a portion of the output signal could be from YFP emission bleeding through into the mCherry channel or from direct mCherry excitation by 488 nm light. The contributions from YFP and the sensitized mCherry emission were determined using viruses labeled with only YFP-Vpr or only mCherry-Vpr. Viruses were allowed to attach to coverslips, washed with PBS^+/+^, and imaged in TIRF mode. The sample was excited at 488 nm and 561 nm with an emission filter for GFP and mCherry, respectively, to emulate live-cell imaging conditions. 

Signal bleed-through between donor (YFP) and acceptor (mCherry) fluorescence was measured using ICY software by quantifying YFP and mCherry channel intensities for single-labeled control viruses and calculating percentage of mCherry signal upon 488 nm excitation compared with 488 nm/YFP or 561 nm/mCherry signals. Normalized FRET (nFRET) was calculated using the equation:

$$nFRET=\frac{I_{488/mCh}-B_{mCH}I_{488/YFP}-B_{YFP}I_{561/mCh}}{I_{561/mCh}}$$

where I_488/mCh_ represents mCherry signal upon excitation with 488 nm, I_488/YFP_ represents YFP intensity, and I_561/mCh_ represents mCherry intensity. The bleed-through contributions are denoted by B_mCH_ for sensitized mCherry emission and B_YFP_ for YFP signal registered in the mCherry channel.

### 4.7. Live-Cell Single-Particle Tracking and Monitoring of Protease Activation

Single-particle tracking was performed for nascent particles forming on the basal cell membrane and particles that were present at the start of imaging and later exhibited a decrease in nFRET signal. First, single-particle tracking was performed using ICY software for preexisting particles and *de novo* forming particles that exhibited a visible increase in YFP signal, while mCherry signal remained constant. Such selective increase in YFP signal is indicative of loss of FRET due to the mCherry-cl-YFP-Vpr cleavage. To ensure that no FRET decrease events were missed due to less apparent increases in the YFP signal, we also tracked random *de novo* appearing particles, irrespective of changes in YFP signal intensity. Fluorescence background subtraction was done in ICY by first obtaining intensity over time for a particle and then subtracting the local background intensity for each frame. 

The nFRET signal was calculated, as described above, and the nFRET curves were fitted to the following sigmoidal function using Excel (dx.doi.org/10.17504/protocols.io.78ihrue):

$$f\left(x\right)=\min+\frac{\max-min}{1+10^{n(\log x-\log T50)}}$$

The times corresponding to 10% and 90% decreases in nFRET (T_10_ and T_90_, respectively) were automatically outputted. This allowed for the determination of the time to protease activation (time from particle appearance to 10% of nFRET decrease, T_PR_) and the time for substrate cleavage defined as the transition time from high (T_10_) to low (T_90_) nFRET, ΔT (see Figure 3B).

To assess the fraction of nascent virions exhibiting a decrease in nFRET, *de novo* forming viral particles were randomly selected, irrespective of any subsequent color change that could be indicative of maturation. Single-particle tracking and nFRET calculations were performed, as described above. Particles were then categorized into decreasing nFRET, constant low nFRET (<0.25), and constant high nFRET (>0.25).

### 4.8. Statistical Analysis

A Mann–Whitney test or a Fisher’s Exact test were used to assess statistical significance in GraphPad Prism (version 10.5.0), unless otherwise stated.

## Data Availability

All pertinent data are included in the main and Supplementary Figures.

## Supplementary Materials

The following supporting information can be downloaded at: https://www.mdpi.com/article/10.3390/v18080819/s1. Figure S1: Example of PR activation in single nascent viruses formed on 293T cells; Figure S2: An isolated PR activation event in a nascent pseudovirus produced the presence of SQV; Figure S3: A visually undetectable PR activation event revealed by single-particle tracking; Figure S4: nFRET value distribution for single pseudoviruses assembled in the presence of SQV; Figure S5: Example of a particle exhibiting a stably low initial nFRET throughout single-particle tracking; Figure S6: Analyses of PR activity in nascent virions containing the mCherry-2xcl-YFP-Vpr substrate; Figure S7: Kinetic analysis of PR activity in nascent virions produced using a different ratio of HIV-1 backbone to FRET substrate ratio; Figure S8: Analysis of FRET substrate cleavage in cell-free virions; Figure S9: Extent of FRET substrate processing assessed by loss of mCherry upon virus lysis; Figure S10: Alternative analysis of mCherry release from saponin-lysed virions; Figure S11: Effect of PF74 on the extent of mCherry loss from virions upon saponin lysis; Figure S12: Western blot analysis of Gag and FRET substrate cleavage in cell-free pseudoviruses; Figure S13: An example of fast substrate cleavage in virions containing the FRET substrate with PR/RT cleavage site; Figure S14: An example of slow cleavage of the FRET substrate with PR/RT cleavage site; Figure S15: The magnitude of nFRET drop in single assembling HIV-1 particles is independent of the PR cleavage sequence; Movie S1: Example of *de novo* forming particle undergoing FRET substrate cleavage; Movie S2: Another example of *de novo* forming particle undergoing FRET substrate cleavage; Movie S3: Example of *de novo* forming particles showing no substrate cleavage.
