# Supplementary material for: Real-Time Imaging of HIV-1 Protease Activation and Substrate Cleavage in Single Virions Assembling on the Plasma Membrane
Source: Viruses. 2026 Jul 25;18(8):819. doi: 10.3390/v18080819 (PMC13517206; doi:10.3390/v18080819)
Supplement: Supplementary file 1 [file viruses-18-00819-s001.zip › viruses-4439875-supplementary-ACSNano_PR activation SupplMaterials.pdf]

Real-Time Imaging of HIV-1 Protease Activation and Substrate Cleavage in Single Virions Assembling on the Plasma Membrane

Yinglin Li[1], Satya P. Singh[2], Mariana Marin[1,3], Alec L. Zhan[1], Ashwanth C. Francis[2], and Gregory B. Melikyan[1,3]

Affiliations:

[1] Department of Pediatrics, Division of Infectious Diseases, Emory University School of Medicine, Atlanta, GA 30322, USA

[2] Institute of Molecular Biophysics and Department of Biological Sciences, Florida State University, Tallahassee, Florida 32306, USA.

[3] Children's Healthcare of Atlanta, Atlanta, GA 30322, USA

Supplementary Materials

Supplemental Figures (S1-S15) and Movies S1-S3

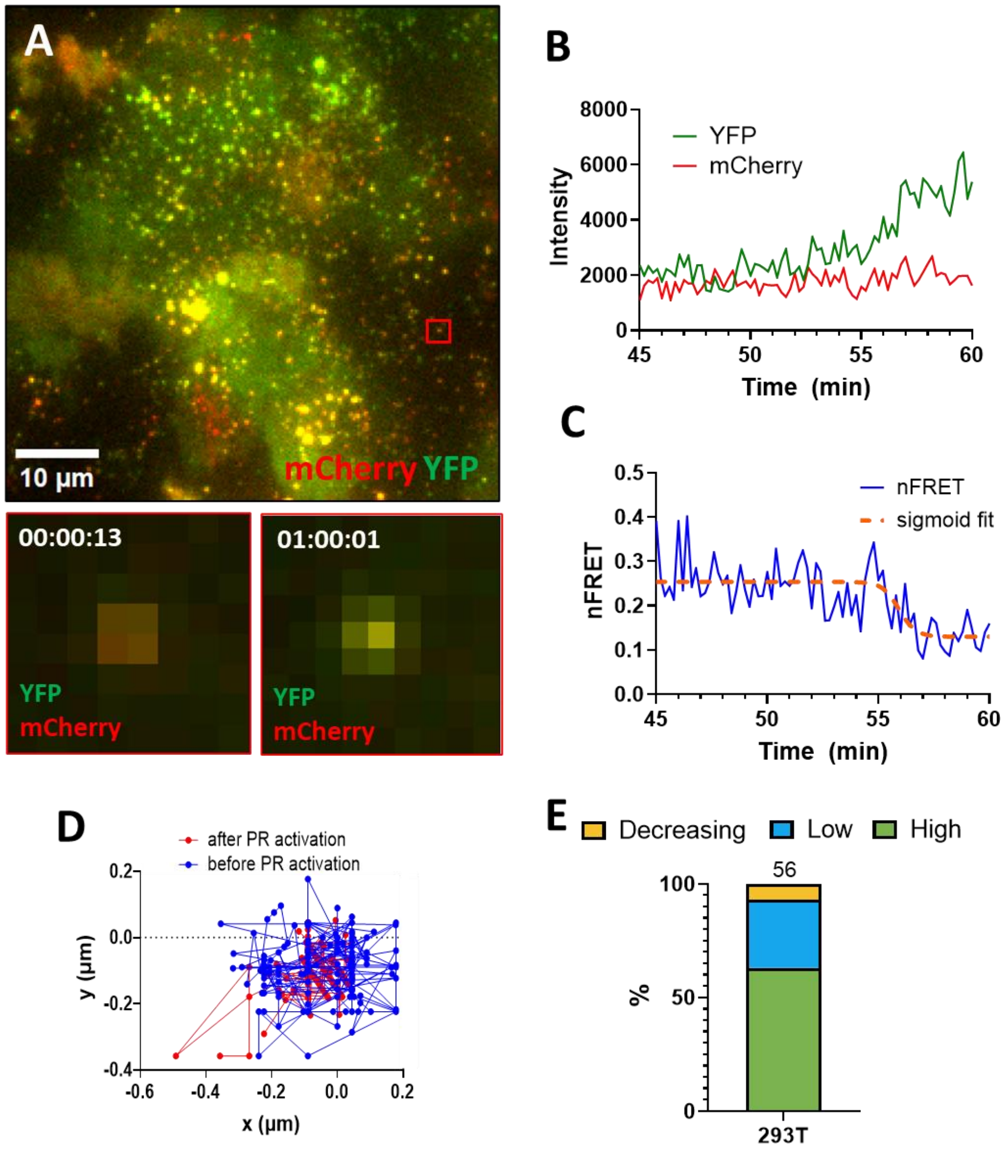

Figure S1. Example of PR activation in single nascent viruses formed on 293T cells. 293T cells were transfected with HIV-1 backbone, HXB2 Env, and mCherry-2xcl-YFP-Vpr, and imaged at 43 hpt using TIRF microscopy. (A) A representative cell is shown. mCherry is red and YFP is green. *Inset:* nascent particle boxed in panel A is shown before (left) and after (right) protease activation (increase in YFP signal). (B) Fluorescence intensity traces of the particle in A, showing an increase in YFP signal. (C) An nFRET trace with a sigmoid fit (dashed line) for the particle shown in A. Time to PR activation is 55.1 min, and the cleavage duration is 1.6 min. (D) XY coordinate trace for the particle in A before and after PR activation. (E) Nascent puncta on live 293T cells were randomly selected and their fluorescence intensities and the nFRET values plotted over time. The nFRET values are categorized into decreasing, low (<0.25), or high (>0.25) nFRET. High/low nFRET cutoff was determined from the nFRET distribution in the presence of SQV.

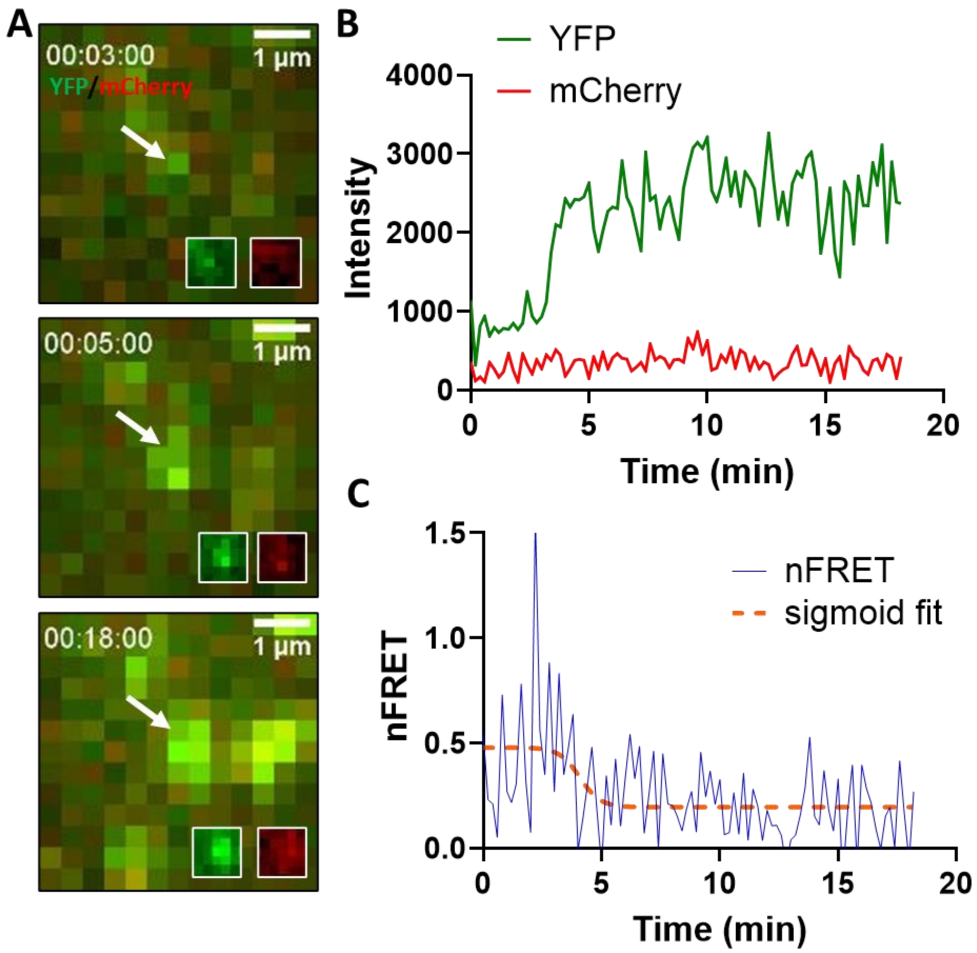

Figure S2. An isolated PR activation event in a nascent pseudovirus produced the presence of SQV. HeLa cells were transfected with HIV-1 backbone, HXB2 Env, and mCherry-2xcl-YFP-Vpr plasmids in the presence of 500 nM SQV and imaged at 43 hpt. (A) Images of a particle showing a decrease in nFRET. (B) Intensity traces corresponding to the particle shown in A. An increase in YFP signal at ~4 min indicates cleavage of the mCherry/YFP FRET pair by HIV-1 PR. (C) nFRET trace for the puncta shown in A. (This isolated particle is included in the analyses shown in Figures 2E and 7B).

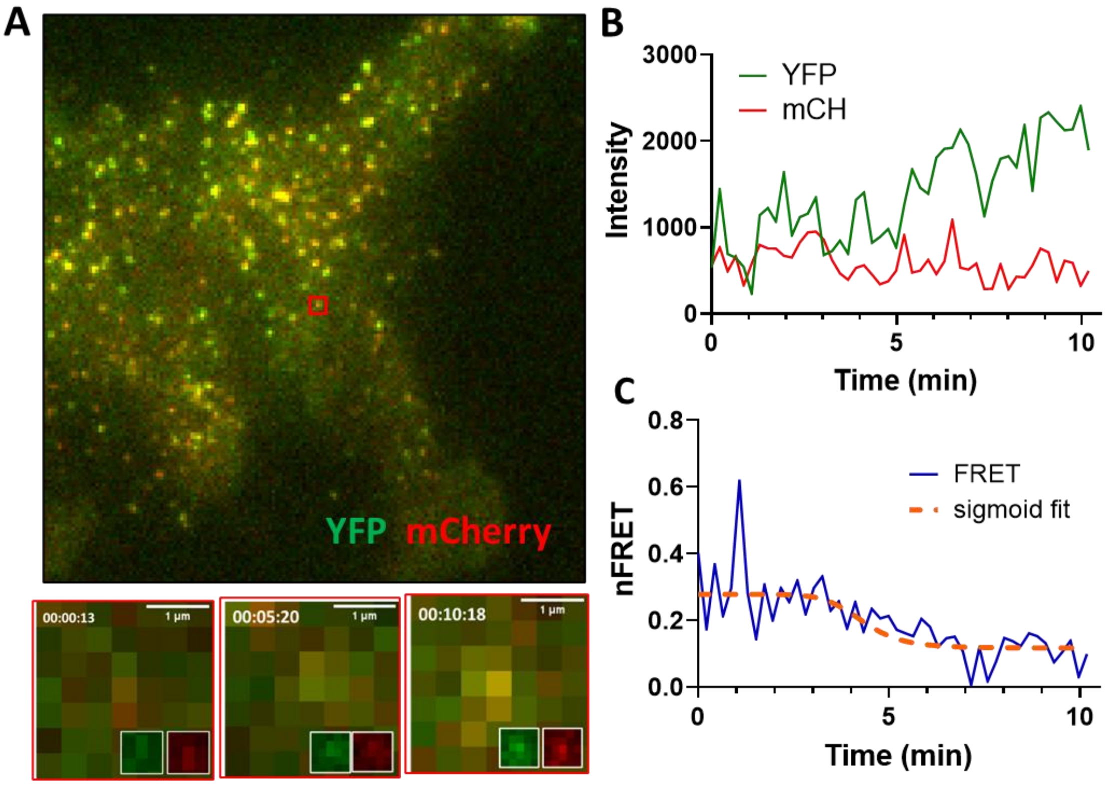

Figure S3. A visually undetectable PR activation event revealed by single particle tracking. HeLa cells were transfected with HIV-1 backbone, HXB2 Env, and mCherry-2xcl-YFP-Vpr plasmids. (A) Representative image of a HeLa cell imaged in a TIRF mode 43 hpt. YFP is green and mCherry is red. *Inset*: Close-up of the particle showing a decrease in nFRET, but no obvious color change (boxed in A). Snapshots at 0, 5 and 10 min after initial appearance are shown. (B) Intensity traces corresponding to the puncta shown in A. (C) nFRET trace for the puncta shown in A, fitted with a sigmoid curve (dashed line).

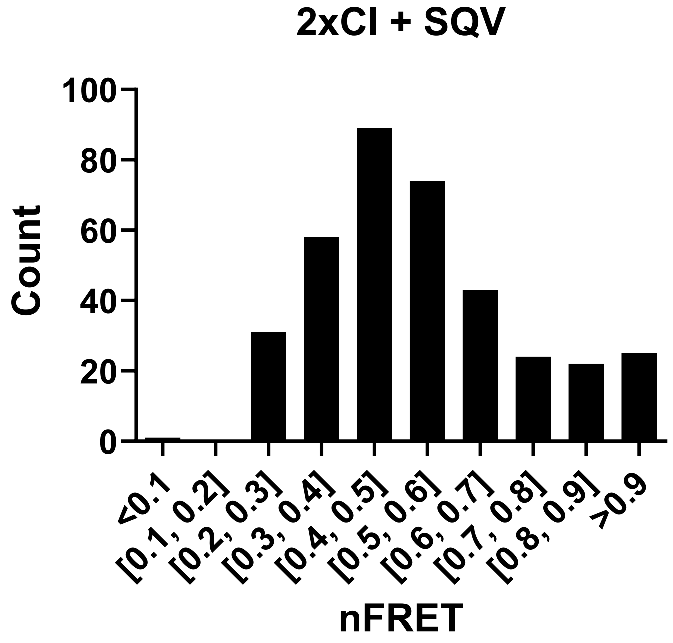

Figure S4. nFRET value distribution for single pseudoviruses assembled in the presence of SQV. HeLa cells transfected with HIV-1 backbone, HXB2 Env, pcRev, and mCherry-CL-YFP-Vpr plasmids were cultured in the presence of 500 nM SQV and imaged 43 hpt in a TIRF mode. N=367 particles assembled at the ventral membrane were analyzed. This distribution served to set the 0.25 cut-off for categorization into high and low nFRET values of *de novo* forming particles in other figures.

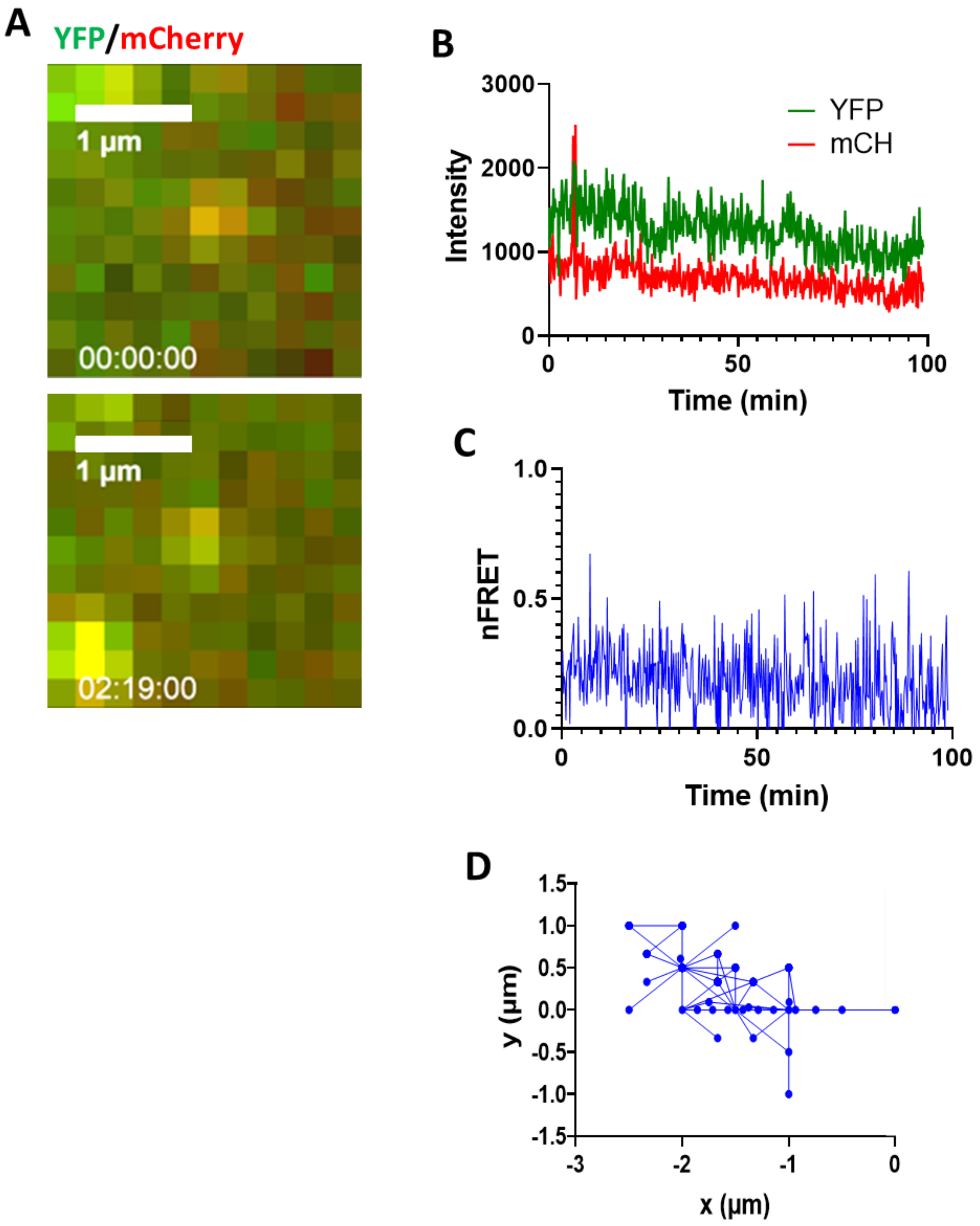

Figure S5. Example of a particle exhibiting a stably low initial nFRET throughout single particle tracking. HeLa cells were transfected with HIV-1 backbone, HXB2 Env, and mCherry-2xcl-YFP-Vpr plasmids. (A) Snapshots of a particle showing no detectable changes in the low nFRET signal at the beginning and end of the experiment. (B) Intensity traces corresponding to the puncta shown in A. (C) nFRET trace for the puncta shown in A. (D) Motion trajectory of the particle in A.

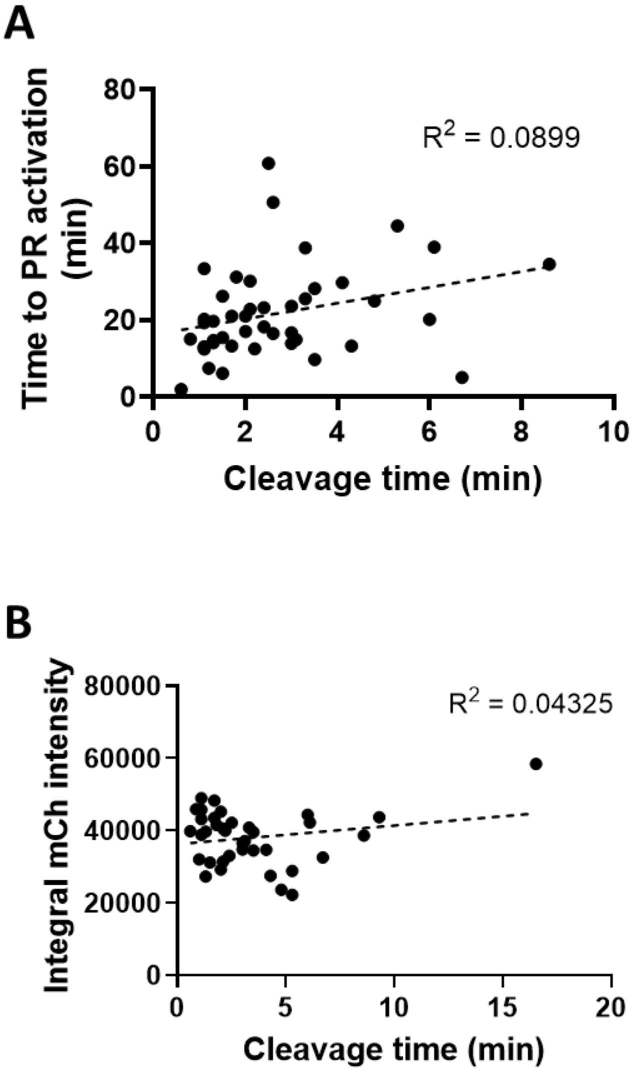

Figure S6. Analyses of PR activity in nascent virions containing the mCherry-2xcl-YFP-Vpr substrate. (A) Scatterplot of substrate cleavage time *vs* the waiting time to PR activation shows no correlation ($R^2 = 0.09$). (B) Scatterplot of substrate cleavage time *vs* the total mCherry intensity per particle before nFRET decrease shows no correlation ($R^2 = 0.04$). Dashed lines are linear regressions.

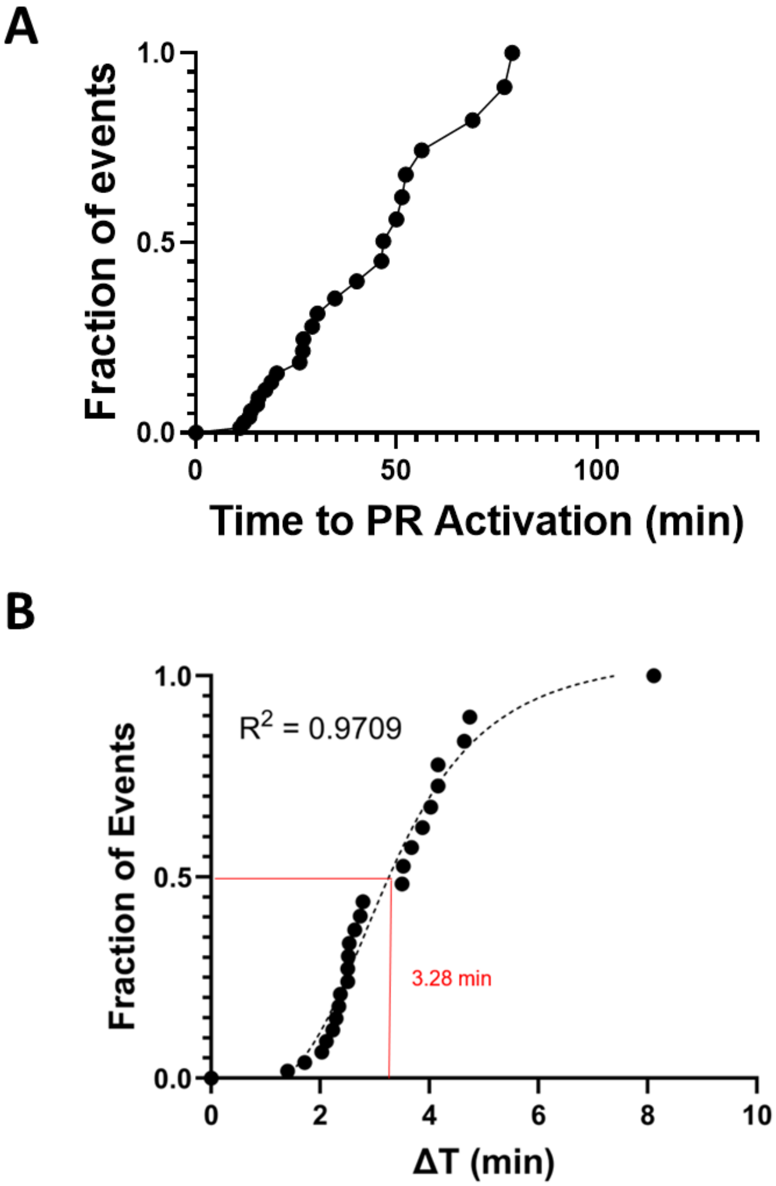

Figure S7. Kinetic analysis of PR activity in nascent virions produced using a different ratio of HIV-1 backbone to FRET substrate ratio. HeLa cells were transfected with a 1:4 ratio of mCherry-2xcl-YFP-Vpr and pR9ΔEnvΔNef plasmids, and the resulting virus assembly and PR activation were imaged in a TIRF mode at 43 hpt. (A) The distribution of waiting times from onset of particle assembly to detectable FRET substrate cleavage (initial decrease in nFRET signal, T_{PR}). (B) The distribution of FRET substrate cleavage times based on the interval for nFRET transitioning from high to low steady state value (ΔT). Dashed line is a sigmoidal fit. (Related to Fig. 3C, D).

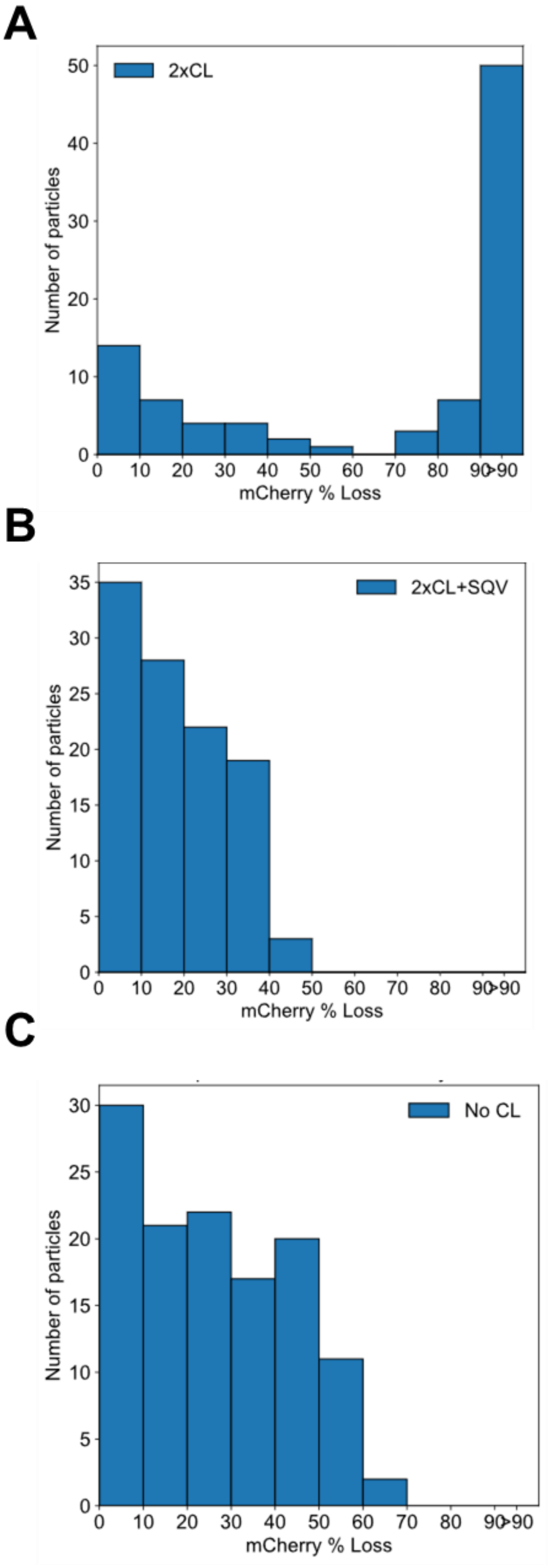

Figure S8. Analysis of FRET substrate cleavage in cell-free virions. HIV-1 pseudoviruses containing mCherry-2xcl-YFP-Vpr were produced from 293T cells in the presence or absence of 500 nM SQV, in parallel with pseudoviruses containing the uncleavable mCherry-YFP-Vpr substrate (No CL). Virions were adhered to a poly-L-lysine coated coverslips and lysed by exposure to 0.1 mg/ml saponin. (A-C) Analysis of the extent of mCherry release from single cell-free virions for 2xcl, 2xcl + SQV and No CL pseudoviruses.

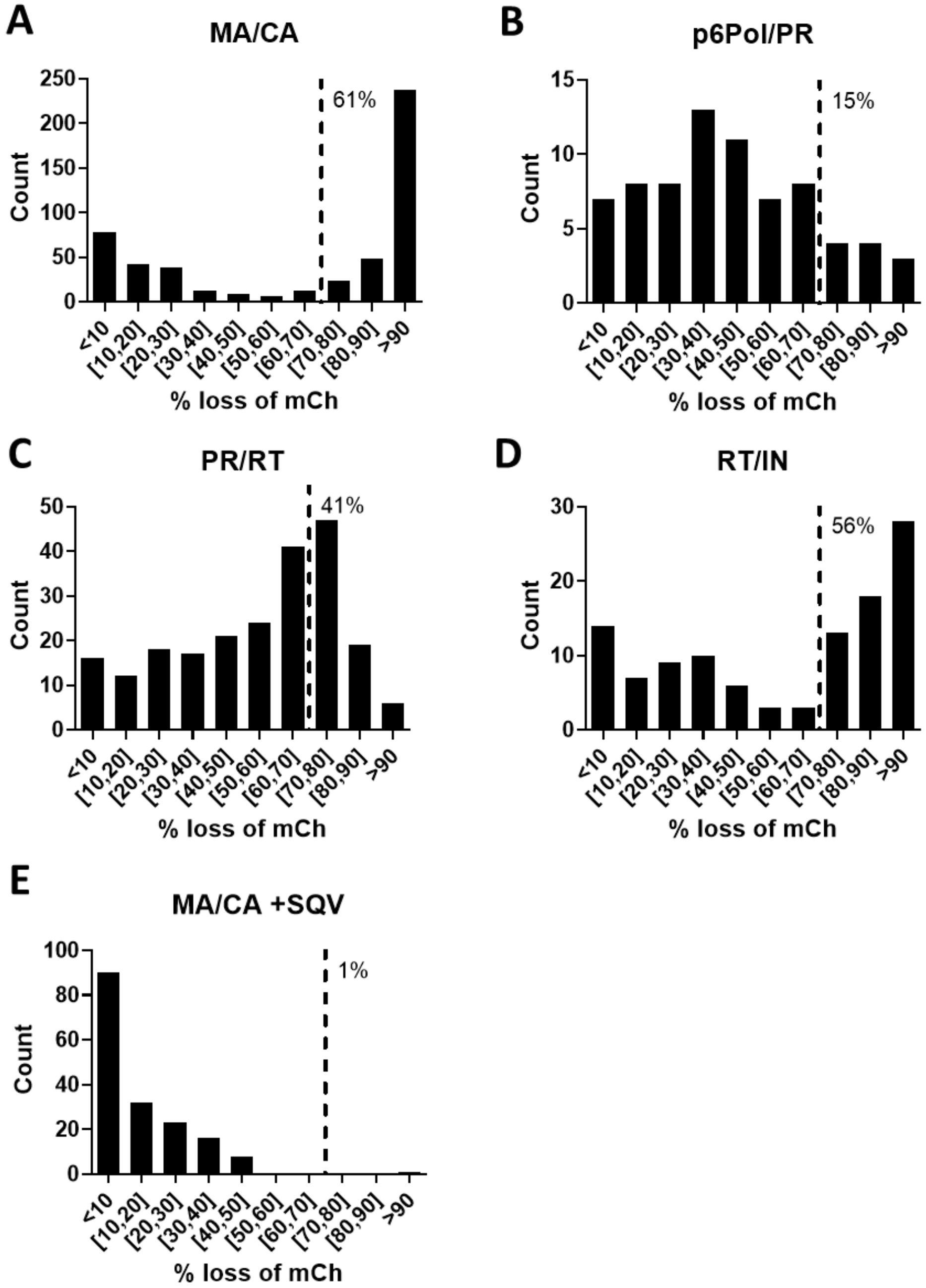

Figure S9. Extent of FRET substrate processing assessed by loss of mCherry upon virus lysis. Pseudoviruses containing FRET substrates with the cleavage sites from MA/CA, RT/IN, p6Pol/PR, or PR/RT were collected from transfected 293T cells and attached to coverslips. The extent of mCherry loss upon lysis with 0.1 mg/mL saponin was measured and plotted as histograms for MA/CA (A), p6Pol/PR (B), PR/RT (C), RT/IN (D), and for immature MA/CA particles produced in presence of SQV (E). Percent particles losing >70% of mCherry signal (operationally defined as "full" lysis and indicated by a dashed line) is shown on the graphs. Dim particles with mCherry intensity less than 1000 A.U. above background were excluded from analysis. (Related to Fig. 5E).

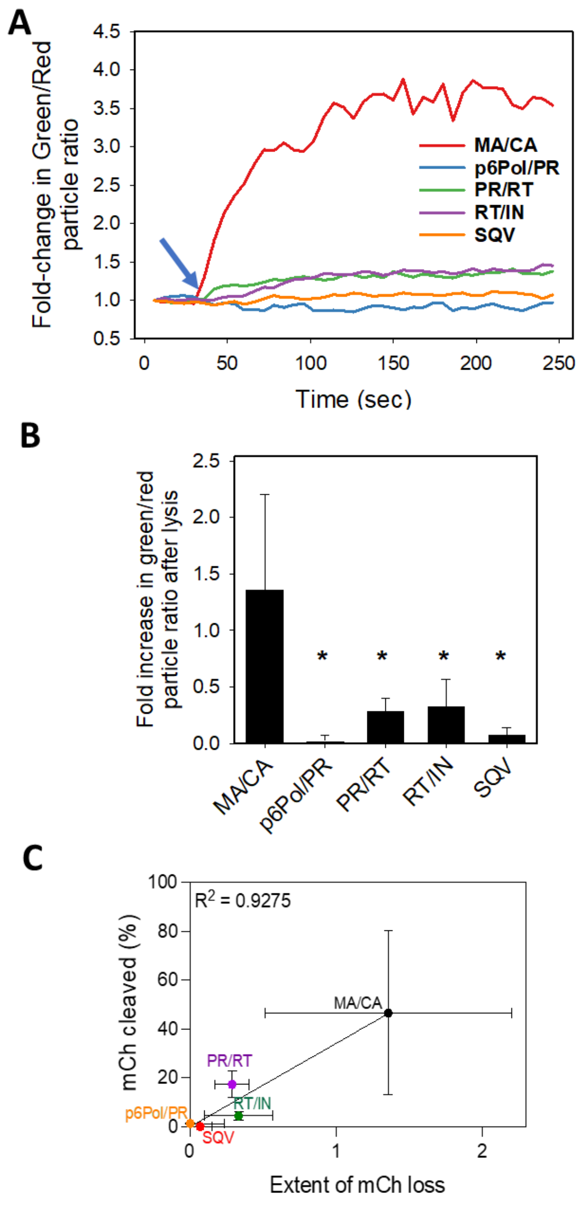

Figure S10. Alternative analysis of mCherry release from saponin-lysed virions. (A) Analyses of mCherry release for a representative virus panel upon exposure to 0.1 mg/mL saponin (blue arrow marks the time of saponin addition). Coverslip-adhered pseudoviruses carrying the FRET substrates with MA/CA, p6Pol/PR, PR/RT or RT/IN cleavage sequences were imaged. Selective loss of mCherry was quantified by calculating fold-change in the ratio of YFP-positive (green) to mCherry-positive (red) particles over time. Ratios below 1 at late times post-lysis are caused by loss of YFP signal exceeding loss of mCherry and/or detachment of lysed virions from a coverslip. (B) Net change in the green/red particle ratio after saponin lysis for 4 independent viral panels. *, $p<0.05$. (C) Correlation between change in green/red ratio after saponin lysis ("Extent of mCh loss") from panel B and FRET substrate cleavage efficiency assessed by Western blotting (see Fig. 6A). Plotted are means and SD for 4 independent preparations. Solid line is linear regression ($R^2=0.93$).

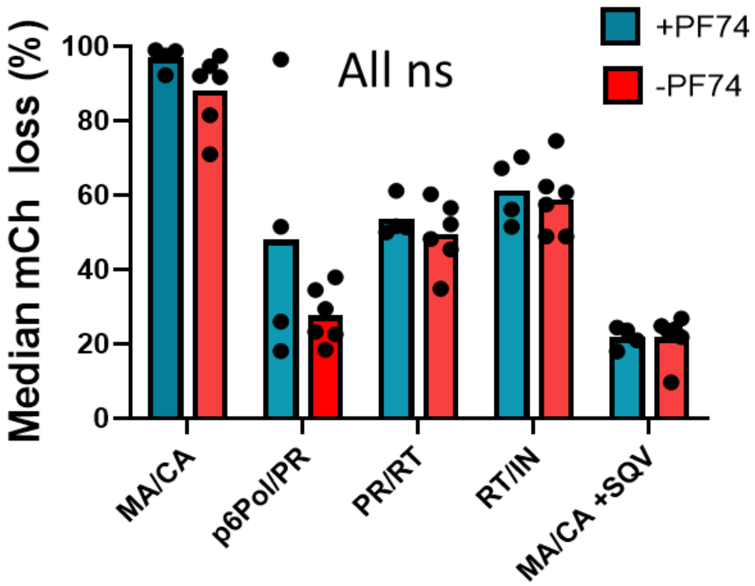

Figure S11. Effect of PF74 on the extent of mCherry loss from virions upon saponin lysis. 293T-produced pseudoviruses were collected and attached to poly-L-lysine coated coverslips. Pseudoviruses were lysed with 0.1 mg/mL saponin in the presence of absence of 20 µM PF74, which is known to disrupt HIV-1 core integrity and cause loss of fluorescent proteins trapped in intact HIV-1 cores. Loss of the mCherry (mCh) signal was monitored by time-lapse imaging and compared for control and PF74-treated particles. Statistical analysis was done using a Mann-Whitney test. No significant effect of PF74 on mCherry release was found.

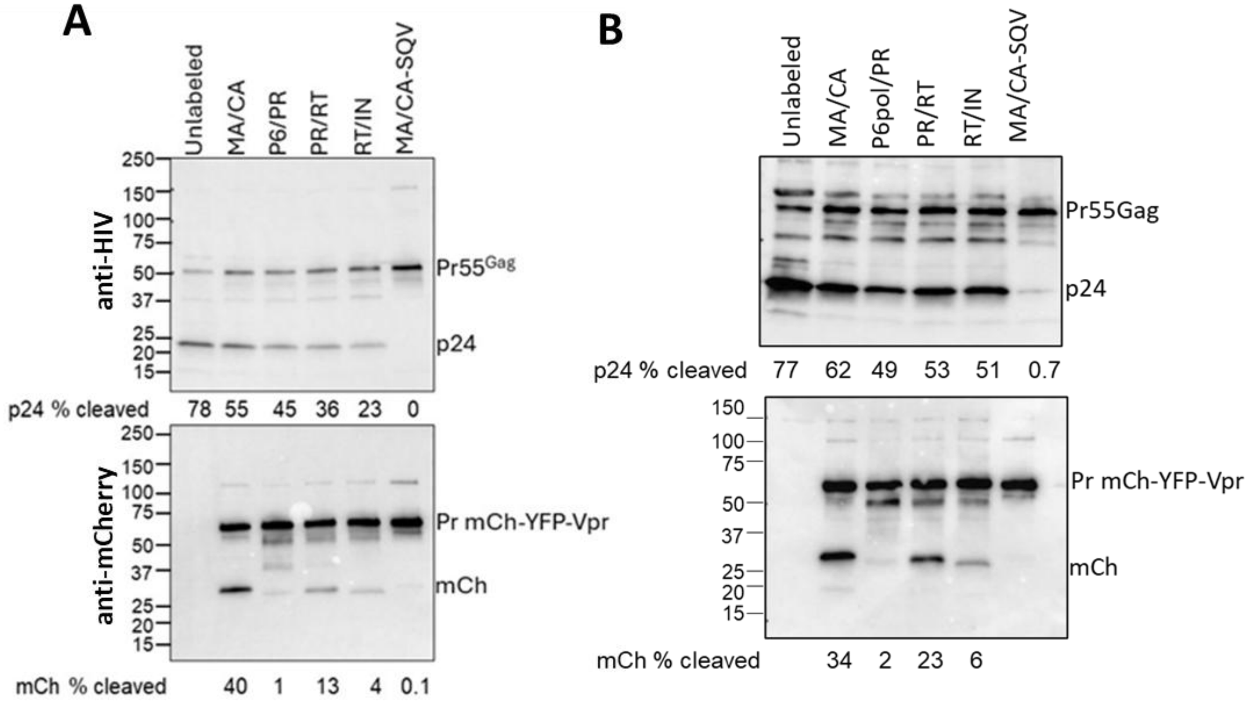

Figure S12. Western blot analysis of Gag and FRET substrate cleavage in cell-free pseudoviruses. (A, B) Immunoblot analysis of two pseudovirus panel produced independently of the one shown in Figure 6A, B, using anti-HIV IG (top blots) or anti-mCherry antibody (bottom blots). Densitometry results of each lane are shown below the blots.

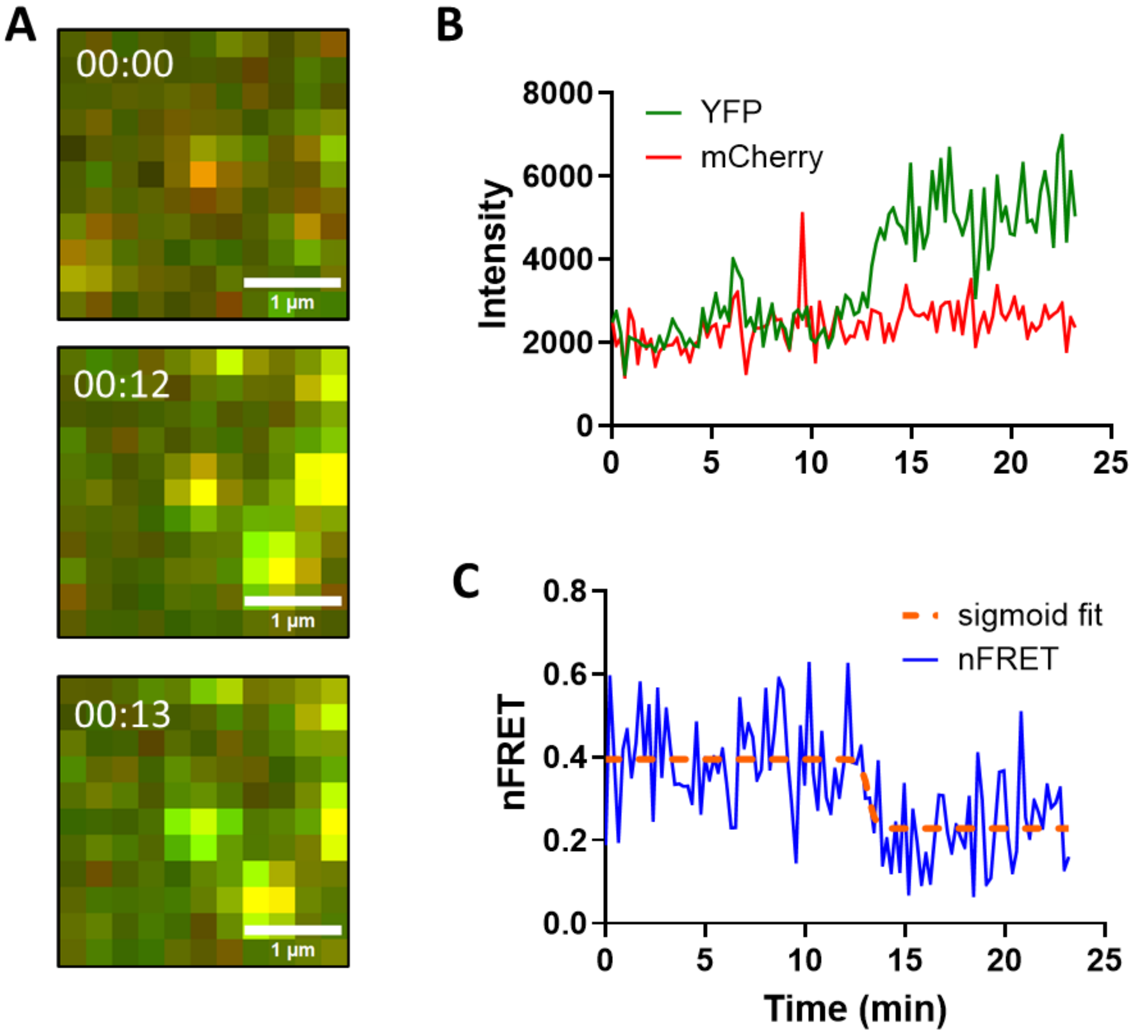

Figure S13. An example of fast substrate cleavage in virions containing the FRET substrate with PR/RT cleavage site. HeLa cells were transfected with HIV-1 backbone, HXB2 Env, mCherry-PR/RT-YFP-Vpr plasmids and imaged at 43 hpt in a TIRF mode. (A) Time-lapse images of a representative particle exhibiting a fast increase in YFP signal indicative of PR activity. (B) Fluorescence intensity traces of the particle in panel A. (C) nFRET trace with a sigmoid fit (dashed line). Based on the curve fit, the waiting time for PR activation was determined to be 12.8 min and the substrate cleavage duration was 1.2 min.

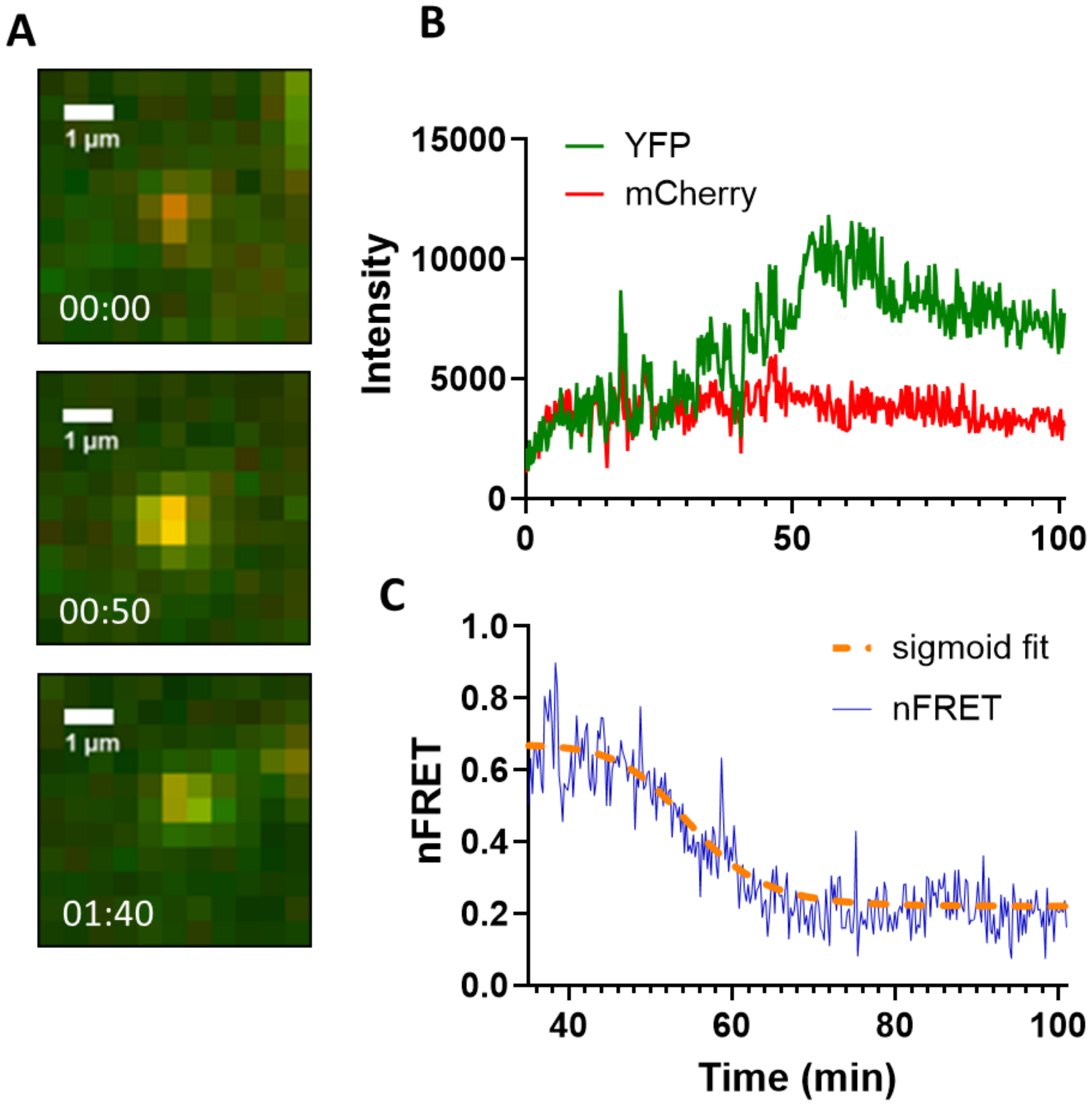

Figure S14. An example of slow cleavage of the FRET substrate with PR/RT cleavage site. HeLa cells were transfected with HIV-1 backbone, HXB2 Env, mCherry-PR/RT-YFP-Vpr plasmids and imaged at 43 hpt in a TIRF mode. (A) Close-up of the particle showing a slow decrease in nFRET at 0, 50, and 100 min after appearance. (B) Fluorescence intensity traces of the particle in (A). (C) nFRET trace with a sigmoid fit (dashed line) for the particle in (A). Based on the curve fit, the waiting time for PR activation was determined to be 46 min and the substrate cleavage duration was 19 min.

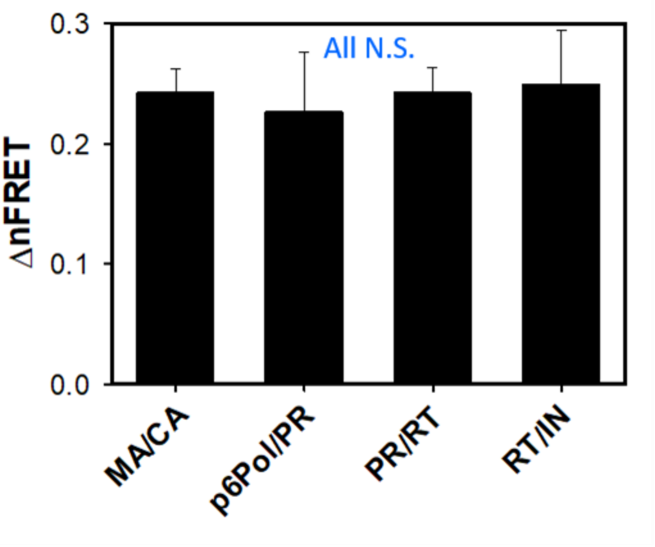

Figure S15. The magnitude of nFRET drop in single assembling HIV-1 particles is independent of the PR cleavage sequence. The PR activation events in particles assembling on the ventral membrane of HeLa cells (see Fig. 7) were analyzed for the magnitude of nFRET drop as a function of the PR cleavage sequence in the FRET substrate. Statistical analysis was done using a Student's t-test.

Movie legends

Movie S1. Example of *de novo* forming particle undergoing FRET substrate cleavage. HeLa cells were transfected with HIV-1 backbone, HXB2 Env, and mCherry-2xcl-YFP-Vpr plasmids and imaged at 43 hours post-transfection in a TIRF mode. YFP is colored green and mCherry is red. A representative particle (corresponding to Figure 1B) showing change in the apparent color from orange to greenish is shown.

Movie S2. Another example of *de novo* forming particle undergoing FRET substrate cleavage. HeLa cells were transfected with HIV-1 backbone, HXB2 Env, and mCherry-2xcl-YFP-Vpr plasmids and imaged at 43 hours post-transfection in a TIRF mode. YFP is colored green and mCherry is red. A representative particle showing a selective increase in YFP signal, a hallmark of FRET reduction, is shown.

Movie S3. Example of *de novo* forming particles showing no substrate cleavage. HeLa cells transfected with HIV-1 backbone, HXB2 Env, and mCherry-2xcl-YFP-Vpr plasmids were imaged at 43 hours post-transfection in a TIRF mode. YFP is green and mCherry is red. Shown are several particles adjacent to the one exhibiting a decrease in nFRET in Movie 1.
